# Sortase A-mediated crosslinked short-chain dehydrogenases/reductases as novel biocatalysts with improved thermostability and catalytic efficiency

**DOI:** 10.1038/s41598-017-03168-z

**Published:** 2017-06-08

**Authors:** Kunpeng Li, Rongzhen Zhang, Yan Xu, Zhimeng Wu, Jing Li, Xiaotian Zhou, Jiawei Jiang, Haiyan Liu, Rong Xiao

**Affiliations:** 1 Key Laboratory of Industrial Biotechnology of Ministry of Education & School of Biotechnology, Jiangnan University, Wuxi, 214122 P. R. China; 2 National Key Laboratory for Food Science, Jiangnan University, Wuxi, 214122 P. R. China; 3 Center for Advanced Biotechnology and Medicine, Rutgers University, Piscataway, NJ 08854 USA; 4 School of Biotechnology, Jiangnan University, 1800 Lihu Avenue, Wuxi, 214122 P. R. China

## Abstract

(*S*)-carbonyl reductase II (SCRII) from *Candida parapsilosis* is a short-chain alcohol dehydrogenase/reductase. It catalyses the conversion of 2-hydroxyacetophenone to (*S*)-1-phenyl-1,2-ethanediol with low efficiency. Sortase was reported as a molecular “stapler” for site-specific protein conjugation to strengthen or add protein functionality. Here, we describe *Staphylococcus aureus* sortase A-mediated crosslinking of SCRII to produce stable catalysts for efficient biotransformation. Via a native N-terminal glycine and an added GGGGSLPETGG peptide at C-terminus of SCRII, SCRII subunits were conjugated by sortase A to form crosslinked SCRII, mainly dimers and trimers. The crosslinked SCRII showed over 6-fold and 4-fold increases, respectively, in activity and *k*_cat_/*K*_m_ values toward 2-hydroxyacetophenone compared with wild-type SCRII. Moreover, crosslinked SCRII was much more thermostable with its denaturation temperature (T_m_) increased to 60 °C. Biotransformation result showed that crosslinked SCRII gave a product optical purity of 100% and a yield of >99.9% within 3 h, a 16-fold decrease in transformation duration with respect to *Escherichia coli/*pET-SCRII. Sortase A-catalysed ligation also obviously improved T_m_s and product yields of eight other short-chain alcohol dehydrogenases/reductases. This work demonstrates a generic technology to improve enzyme function and thermostability through sortase A-mediated crosslinking of oxidoreductases.

## Introduction

Optically active alcohols are important building blocks for the synthesis of numerous products in pharmaceutical and fine chemical industries^1, 2^. The asymmetric reduction of prochiral ketones by alcohol dehydrogenases/reductases is among the most straightforward and efficient approaches for preparation of chiral alcohols with theoretical yields of up to 100%. Thermostable alcohol dehydrogenases/reductases are desirable for biotransformation reactions because the use of high temperatures can improve reaction rates and decrease microbial contamination^3^. Several techniques have been developed for the thermostabilisation of proteins by site-directed mutagenesis, including introduction of changes to specific hydrophobic interactions^4^, improvements in surface electrostatic interactions^5^, and introduction of disulfide bonds between Cys residues^6^. However, the key residues involved in these mutation studies are not customisable.

The sortase family of transpeptidases catalyses the sequence-specific ligation of proteins to cell wall of Gram-positive bacteria^7, 8^. *Staphylococcus aureus* sortase A (SrtA) (Table 1) specifically recognises LPXTG motifs (where X is any amino acid) and cleaves the Thr-Gly peptide bond, yielding a thioacyl intermediate. The intermediate is attacked by the amino group of the (Gly)_n_. At this time, another Thr-Gly bond is formed and SrtA enzyme is liberated. Sortase-mediated ligation has become a popular technique for the synthesis of protein-nucleic acid^9^, protein-lipid^10^, and protein-surface conjugates^11^ and cyclised peptides^12^. Wu *et al*. used SrtA for chemoenzymatic synthesis of intramolecular head-to-tail bifunctional peptides and glycopeptides^13^. Antos *et al*. described sortase-catalysed transpeptidation as a route to circular proteins with enhanced resistance to denaturation compared with linear counterparts^14^.

**Table 1:** Enzymes in this work.

Enzyme	GeneBank ID	Source	Ref
SrtA	KII20095	*Staphylococcus aureus* ST541	17
SCRII	GQ411433	*Candida parapsilosis* CCTCC M203011	16
ADHR	AY267012	*Lactobacillus kefiri* DSM 20587	29
C1	AB084515	*Candida parapsilosis* IFO 0708	43
C2	AB084516	*Candida parapsilosis* IFO 0708	43
CR2	AB183149	*Kluyveromyces marxianus* AKU 4588	32
CR4	E59061	*Kluyveromyces aestuarii* DC 6752	33
S1	AB036927	*Candida magnoliae* AKU 4643	44
SCR1	FJ939565	*Candida parapsilosis* CCTCC M203011	35
SCR3	FJ939564	*Candida parapsilosis* CCTCC M203011	35

**Figure 1:**
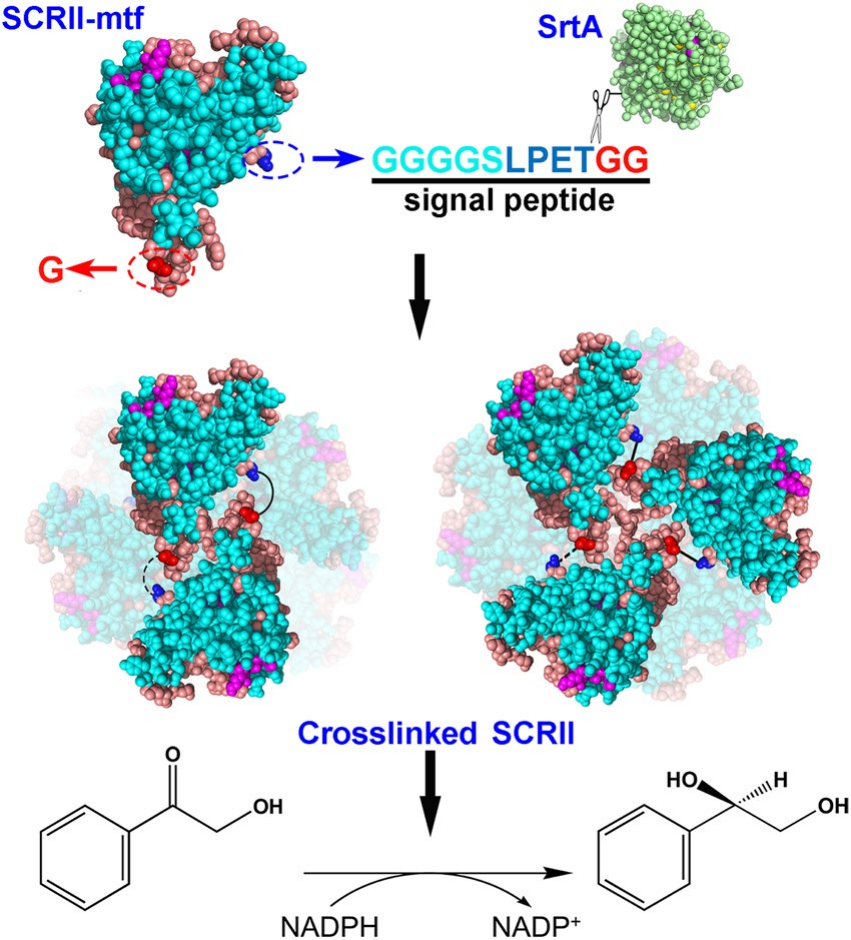
Schematic illustration showing the SrtA-mediated crosslinking of SCRII. GGGGSLPETGG tag was fused into the C-terminal end of SCRII. SrtA recognized and cleaved the tag, followed by the attack from the glycine at the N-terminal end to form crosslinked protein complexes (mainly dimers and trimers). The covalently bond dimers and trimers were used as units to form further oligomerisation, of which the intermolecular conformation was assumed in this work. The dotted line meaned possible covalent bond to form cyclic crosslinked SCRII. The crosslinked SCRII was subsequently applied to the asymmetric reduction of 2-HAP to (*S*)-PED.

We previously reported an NADPH-dependent (*S*)-carbonyl reductase (SCR) from *Candida parapsilosis* CCTCC M203011^15^. It is a member of the short-chain dehydrogenases/reductases family with determined crystal structure as tetramer in solution. Recently, its isozyme, the other novel (*S*)-carbonyl reductase SCRII was employed to catalyse the asymmetric reduction of 2-hydroxyacetophenone (2-HAP) to (*S*)-1-phenyl-1,2-ethanediol ((*S*)-PED) in 48 h (Fig. 1)^16^. However, low thermostability and poor catalytic activity would limit future biocatalytic application of SCRII. Thus, further research is urgently required to undertake protein engineering of SCRII to produce stable catalyst with improved catalytic performance.

Herein, we report for the first time SrtA-mediated crosslinked short-chain dehydrogenases/reductases as robust biocatalysts for efficient chiral synthesis. We carried out sortase-mediated ligation inside SCRII to generate artificial crosslinked protein. The flexible motif GGGGSLPETGG was fused to the C-terminus of SCRII to generate SCRII-GGGGSLPETGG. Via a native glycine at the N-terminus of SCRII, protein ligations between SCRII-GGGGSLPETGG subunits were achieved using *S. aureus* SrtA as catalyst (Fig. 1). The crosslinked SCRII showed significantly improved functionalities and remarkably improved thermal stability. Furthermore, the crosslinked SCRII also stimulated significant increases in yield of (*S*)-PED, and simultaneously reduced the reaction time by 16-fold compared to *E. coli*/pET-SCRII. Eight other short-chain dehydrogenases/reductases (SDRs; Table 1) were selected to construct the corresponding crosslinked oxidoreductases using SrtA-mediated method to realise efficient chiral catalysis. This work provides a new platform of redesigning oxidoreductases to enhance enzyme functionality for efficient and stable chiral biosynthesis.

## Results and Discussion

### SrtA-mediated ligation of SCRII to form crosslinked SCRII

A suitably flexible GGGGS motif was fused to the sorting signal LPETGG that can be recognised by SrtA. The resulting GGGGSLPETGG motif was added to the C-terminus of wild-type (WT) SCRII to generate SCRII-GGGGSLPETGG which was referred as SCRII-mtf. Ilangovan *et al*. confirmed the core domain of SrtA (amino acids 60–206) exhibited the same activity as wild-type sortase^17^. In this work, the truncated SrtA protein was overexpressed and purified with an estimated yield of about 50 mg/L culture and purity of >90% (Supplementary Fig. S1).

**Figure 2:**
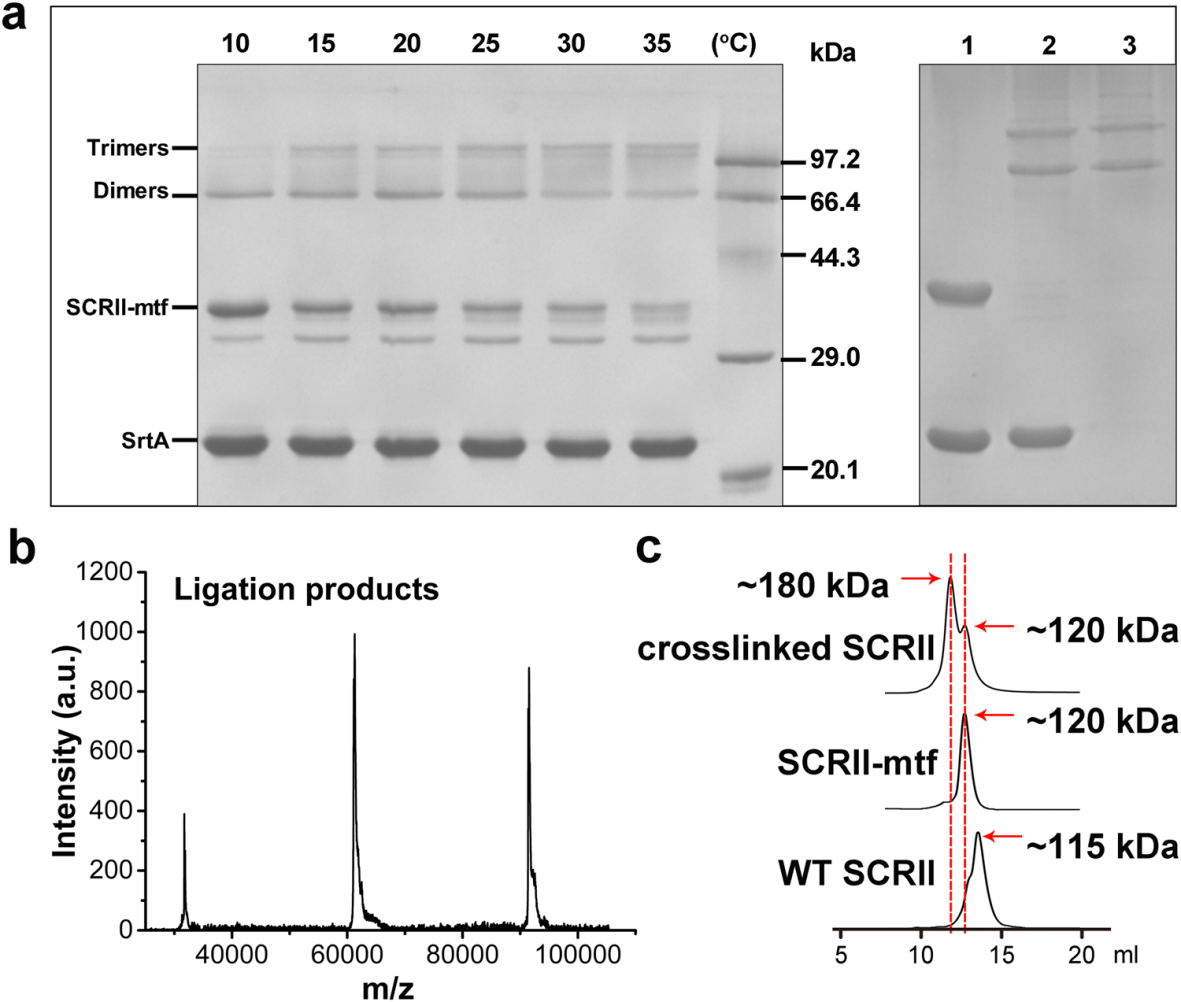
Determination of SrtA-mediated ligation products. (**a**) Left: Optimization of the temperature for the SrtA-mediated ligation. SCRII-mtf and SrtA were incubated at different temperatures for 8 h. Right: Ligation result after 36 h for WT SCRII and SCRII-mtf. Lanes 1 and 2, WT SCRII and SCRII-mtf were incubated with SrtA for 36 h at 25 °C, respectively. Lane 3, Purified crosslinked SCRII. (**b**) MALDI-TOF-MS analysis of crosslinked SCRII. (**c**) The elution profiles of crosslinked SCRII, SCRII-mtf and WT SCRII by size exclusion chromatography. The red dotted lines were used for alignment of peaks.

The SrtA-mediated ligation was initiated by the addition of Ca^2+^ to a mixture of purified SCRII-mtf (~30 kDa) and SrtA (~23 kDa). To determine the optimal temperature for SrtA-catalysed crosslinking of SCRII, the mixture was incubated at temperatures in the range 10–35 °C for 8 h. SDS-PAGE analysis revealed the presence of two major bands corresponding to the expected sizes of dimers and trimers of SCRII (about 66 and 99 kDa) (Fig. 2a). Matrix assisted laser desorption/ionisation-time of flight mass spectrometry (MALDI-TOF-MS) analysis further confirmed the appearance of dimers and trimers in the ligation mixture of SCRII (Fig. 2b). Two byproduct bands were also observed below the band corresponding to SCRII-mtf, which were excised for MALDI-TOF-MS. Peptide mass fingerprinting showed that both of them were most like SCRII (Supplementary Fig. S2). According to previous reports^14, 18^, they could be assigned as SCRII-GGGGSLPET and cyclised SCRII-GGGGSLPET. Based on the amount of ligation product, the optimal temperature for the SrtA-mediated ligation was determined to be 25 °C. SDS-PAGE analysis with Bandscan 5.0 revealed that the relative intensity of dimers and trimers accounted for >90% of the ligation products, not including SCRII-mtf and bands below it. As a control, we did not observe any product in SrtA-mediated ligation of WT SCRII.

Interestingly, SCRII-mtf and two byproducts (supposed to be SCRII-GGGGSLPET and cyclised SCRII-GGGGSLPET) were almost completely consumed after 36 h at 25 °C. The behavior was contrary to the results of reports on the SrtA-mediated ligation of eGFP. Antos *et al*. found that bifunctional eGFP strongly favoured cyclisation with little evidence of any oligomerisation^14^. There are two key differences between their experiments and ours: (1) different proteins have different distances between their N- and C-termini. Previous work^15^ revealed the structure of SCR which had 86% similarity to SCRII. The distance between N- and C-termini was not close, which reduced the chance of cyclisation occurring; and (2) SrtA-catalysed cyclisation is a reversible reaction^19^. The cleavage of a cyclised subunit would generate a thioacyl intermediate which could be attacked by the N-terminal glycine of another protein molecule to form a crosslinked complex. In contrast, the cross-linked SCRII would be difficult to be cleaved by SrtA because of steric hindrance of the LPETG motif inside the complex.

To remove SrtA and residual SCRII-mtf from SrtA-mediated ligation products, the ligation mixtures were subjected to size exclusion chromatography. As shown in Fig. 2c, the crosslinked SCRII were corresponding to two peaks in the range of 120 kDa–180 kDa. These results indicated that the covalently-linked dimers and trimers formed tetramers and hexamers. Besides, the covalently-linked dimer and trimer were found in both of the two peaks. We inferred that a dynamic equilibrium existed between the tetramer and hexamer. For example, two hexamers may consist of six dimers which could be recombined into three tetramers. The process was reversible, dynamic and happening all the time in solution. Therefore, the products cannot be fully separated. SCRII-mtf and WT SCRII had narrow molecular mass distributions with a single peak observed at ~120 kDa and ~115 kDa (Fig. 2c), suggesting they both formed a tetramer in solution. Our previous work revealed that SCR, the isoenzyme of SCRII, existed as tetrameric form in solution. We conclude that SCRII-mtf and WT SCRII formed similar tetrameric conformations to each other. However, the conformation of the tetramers and hexamers formed by crosslinked SCRII could not be determined.

### SrtA-mediated crosslinked SCRII has improved thermostability without secondary structural changes

**Figure 3:**
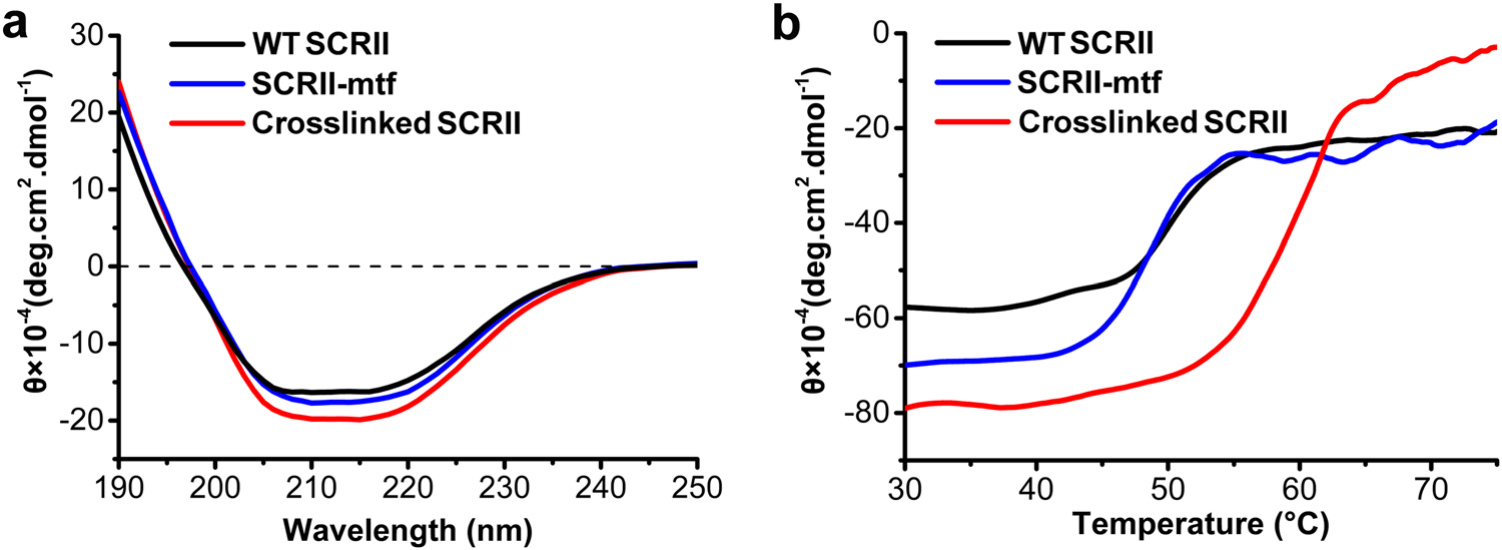
CD spectra (**a**) and thermal shift assays (**b**) of WT SCRII, SCRII-mtf and crosslinked SCRII. The CD spectra were recorded by measuring the ellipticity as a function of wavelength at 0.1-nm increment between 190 and 250 nm at 20 °C. Thermal shift assay was conducted by heating the three samples from 20 to 90 °C and then cooling to 20 °C by a Jasco programmable Peltier element with a rate of 1 °C/min. The wavelength of 209 nm that characterized the α-helix within protein was used to monitor the unfolding rate of the protein structures. The buffer (5 mM phosphate buffer pH 6.0, 0.1 mg/mL enzymes.) showed pH shift with no more than 0.5 while it was heated from 20 °C to 100 °C.

The purified crosslinked SCRII, SCRII-mtf and WT SCRII were analysed by far-UV circular dichroism spectroscopy (Fig. 3a) to determine whether any change occurred in secondary structure content. All three samples showed a positive and a negative band around 190 and 208 nm which are characteristic of an α-helical structure^20–22^. The deconvolved data showed that the crosslinked SCRII, SCRII-mtf and WT SCRII enzymes shared similar secondary structures (see Supplementary Table S1), indicating that no significant intramolecular conformational changes were induced by the SrtA-mediated crosslinking of SCRII or introduction of the GGGGSLPETGG tag at its C-terminus. There are no sufficient data to determine whether intermolecular conformational changes occurred.

The thermal stabilities of the three proteins were measured by monitoring the disappearance of their characteristic α-helical peak at 208 nm. Figure 3b showed the thermal shift curves of crosslinked SCRII, SCRII-mtf and WT SCRII. The denaturation temperatures (T_m_) of these proteins were obtained by calculating the maximum slopes of their denaturation curves, also known as the denaturation midpoint^23^. The secondary structures of WT SCRII and SCRII-mtf progressively disappeared at temperatures >45 °C. There was no obvious difference between T_m_s of WT SCRII and SCRII-mtf (50 °C), suggesting the introduced C-terminal tag had little effects on enzyme thermostabilily. In contrast, the secondary structure of crosslinked SCRII remained intact up to 55 °C and then rapidly disappeared, with a T_m_ of 60 °C. Therefore, our attempt to construct SrtA-mediated protein conjugates enhanced the protein thermal stability. Protein thermal stability may be associated with higher α-helix content^24^ while our examination of secondary structures suggested that is not the explanation here. Clantin *et al*. reported that oligomerisation could help achieve thermal stabilisation of a protein, but that oligomerisation was of proteins assembled together to form an interface without covalent linkages between monomers^25^. The improved thermal stability in our work may be attributed to the covalent bonds between SCRII-mtf subunits, which might help to reinforce the structure of the complexes. Popp *et al*. demonstrated the cyclisation of four-helix bundle cytokines by covalently joining the N- and C-termini of the proteins with a resulting improvement in thermal stability^26^. Here, the dimers and trimers may also exist in cyclic forms which could increase thermal stabilisation by possible formation of salt bridges and the consolidation of protein structure, though this speculation remains to be assessed in future research.

### Crosslinked SCRII has significantly improved chiral synthesis efficiency compared with WT SCRII

**Figure 4:**
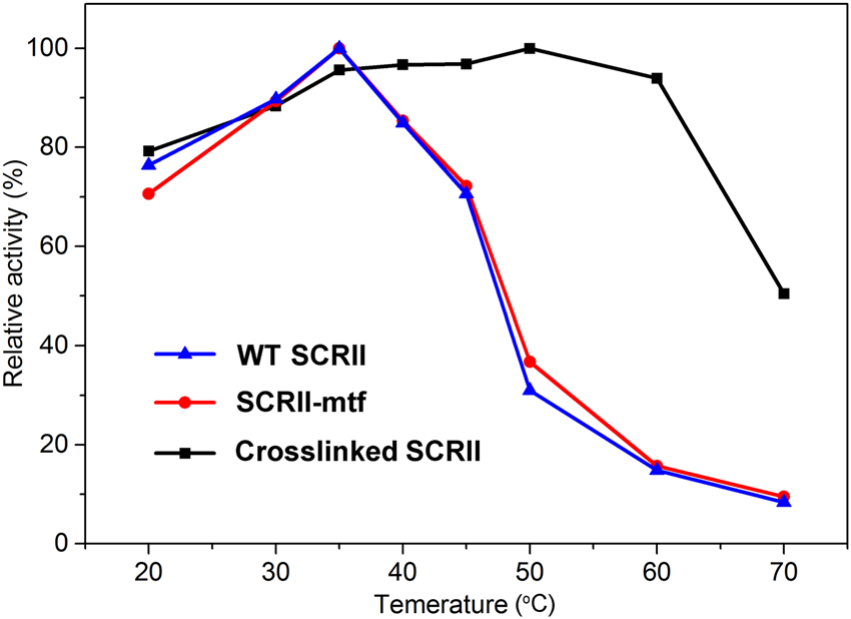
Effects of temperature on enzyme activity for WT SCRII, SCRII-mtf and crosslinked SCRII. Enzyme assay was performed in 100 mM potassium phosphate buffer (pH 6.0), 0.5 mM NADPH, 5 mM substrate, and appropriate enzymes (mg/mL) at 20–70 °C. The relative activity was described as percentage of maximum activity under experimental conditions. All experiments were repeated three times.

**Figure 5:**
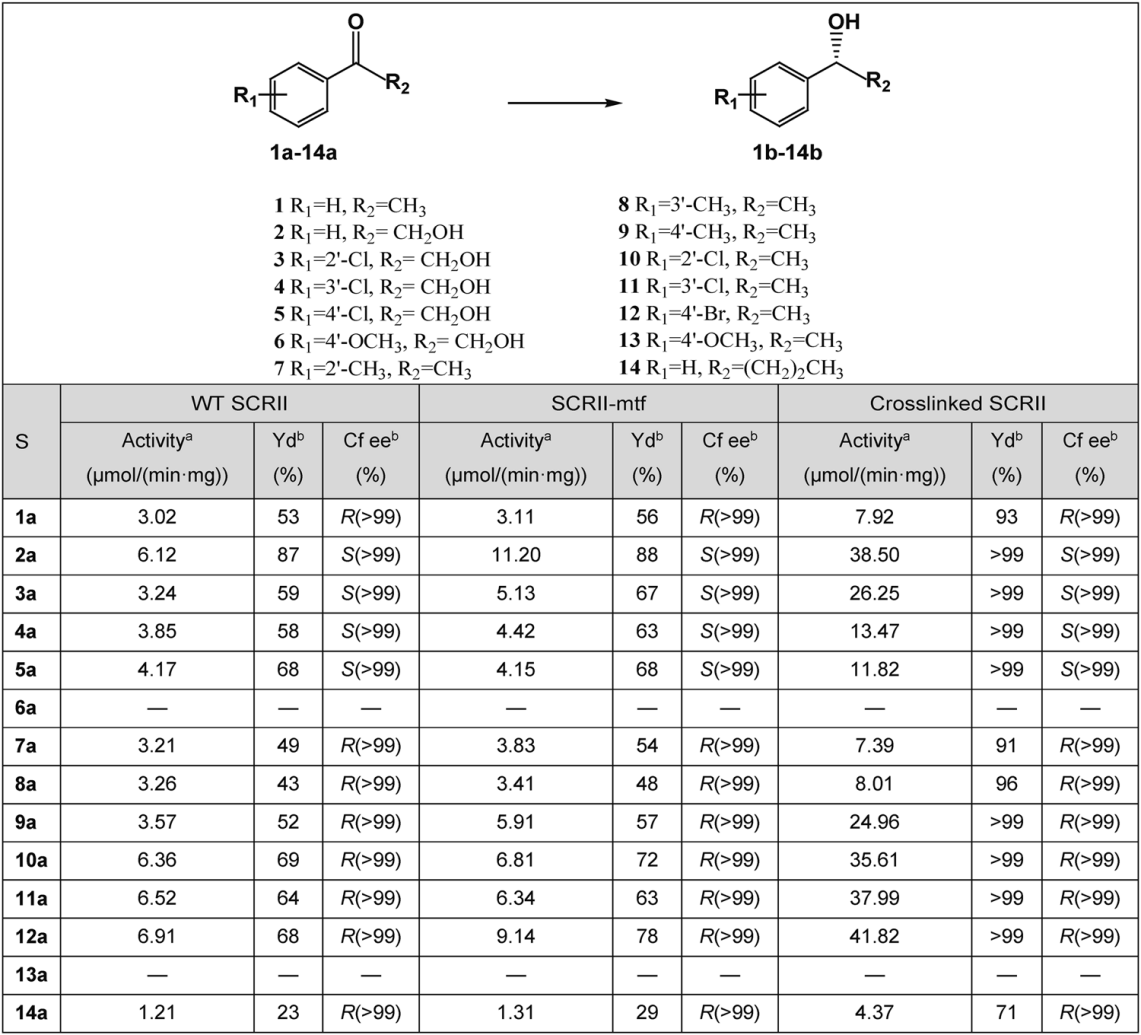
Substrate specificities of WT SCRII, SCRII-mtf and SrtA-mediated Crosslinked SCRII. ^a^Standard assay conditions: 100 mM phosphate buffer (pH 6.0), 0.5 mM NADPH, 5 mM substrate, 1 mg/mL enzymes at 35 °C. The data was the representative of three independent experiments and standard errors were not more than 5%. ^b^The biotransformation was conducted with 5 g/L substrate and sufficient NADPH for 6 h. S, substrate; Yd, yield; Cf, configuration, ee, enantiomeric excess.

We previously reported that WT SCRII enzymes showed the highest specific activity at 35 °C^16^. Here, the optimal reaction temperature for crosslinked SCRII toward 2-HAP was determined from 20 °C to 70 °C. Meanwhile, WT SCRII and SCRII-mtf enzymes were tested as controls. As shown in Fig. 4, the crosslinked complexes showed their highest reductive activity at 50 °C. At 35 °C, the crosslinked SCRII showed a specific activity of 38.50 μmol/(min·mg), about 95% of the maximum observed activity and 6.2-fold higher than that of WT SCRII (Fig. 5). To determine the shortest time needed for the biotransformation of 2-HAP, sampling was performed at various times with crosslinked SCRII, SCRII-mtf and WT SCRII as catalysts. Crosslinked SCRII completed the asymmetric reduction of 2-HAP to (*S*)-PED with an optical purity of >99% and a yield >99% within 3 h (Supplementary Fig. S3). SCRII-mtf and SCRII showed similar performance to each other, with only about 55% yield in 3 h and 88% yield in 6 h. It was worth mentioning that they could not fully complete the biotransformation, with a maximum yield of about 98%. In addition, compared with whole cell biotransformation by *E. coli*/pET-SCRII^16^, crosslinked SCRII significantly decreased the time for biotransformation of (*S*)-PED, from 48 h to 3 h. To determine the effects of crosslinked SCRII on the substrate specificity, we investigated the asymmetric reduction of 13 different ketones bearing an aryl group. The specific activity of the crosslinked SCRII protein was around 2- to 8-fold higher than that of WT SCRII and SCRII-mtf proteins, except toward substrates **6a** and **13a**. The biotransformation results revealed that crosslinked SCRII improved yields by 13.8–209% compared with WT SCRII, without any reduction in the enantiomeric excess (*ee*) values of the chiral products. The crosslinked SCRII, SCRII-mtf and WT SCRII proteins showed no specific activity in the asymmetric reduction of substrates **6a** or **13a**. These results suggested that the SrtA-mediated conjugation did not change the essential enzyme function, but improved it.

### Kinetics demonstrate increased affinity between crosslinked SCRII and 2-HAP

**Figure 6:**
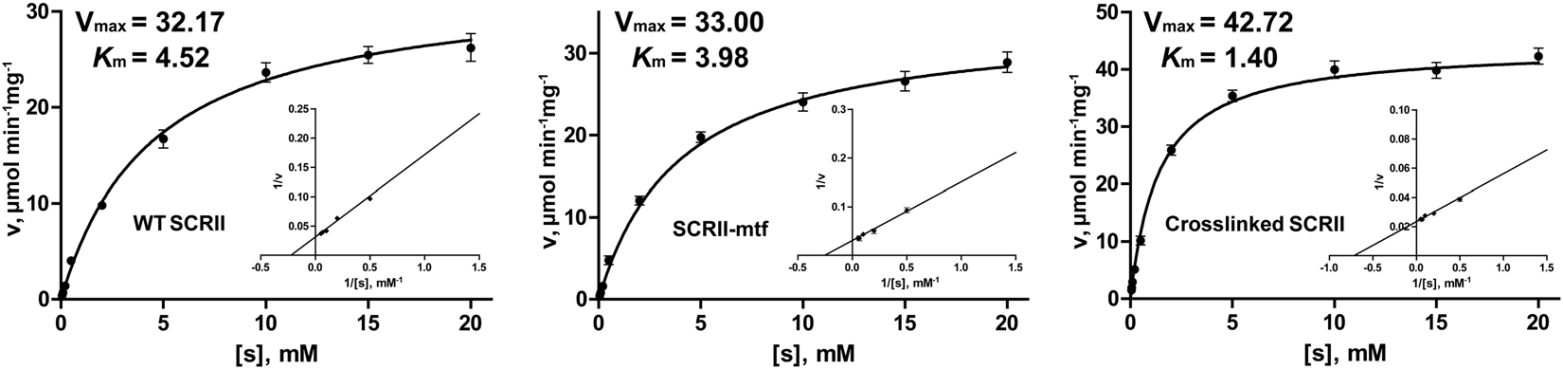
Michaelis-Menten and Lineweaver-Burk plots of WT SCRII, SCRII-mtf and Crosslinked SCRII toward 2-HAP. Each value was calculated depending on three independent measurements and all standard errors of fits were not more than 5%. The data obtained were fitted to the function v = V_max_[s]/(*K*_m_ + [s]).

WT SCRII enzymes favour NADPH as a cofactor over NADH^16^. Therefore, the kinetic effects of crosslinked SCRII, SCRII-mtf, and WT SCRII were evaluated in the reduction of substrates with NADPH. V_max_ and *K*_m_ values were derived from Michaelis-Menten and Lineweaver-Burk plots (Supplementary Table S2). *k*_cat_ and *k*_cat_/*K*_m_ values are summarised in Table 2. Taking reduction of 2-HAP (substrate **2a**), which is the favoured substrate of WT SCRII, as an example (Fig. 6), the *K*_m_ and *k*_cat_ values of the SrtA-mediated crosslinked SCRII protein were 3.3-fold lower and 1.3-fold higher than those of WT SCRII, respectively. Crosslinked SCRII displayed higher catalytic efficiency than WT SCRII toward all 12 ketone substrates (**6a** and **13a** not included) and the *k*_cat_/*K*_m_ values increased by 1.4-fold (**7a**) to 4.4-fold (**12a**). Meanwhile, SCRII-mtf displayed parallel behaviour with WT SCRII toward the tested substrates, indicating that the C-terminal tag had little impact on the enzyme activity.

**Table 2:** Kinetic parameters for WT SCRII, SCRII-mtf and crosslinked SCRII towaids the asymmertric reduction of aryl ketones.

No	WT SCRII			SCRII-mtf			Crosslinked SCRII		
	*K*_m_ (mM)	*k*_cat_ (s^−1^)	*k*_cat_/*K*_m_ (s^−1^mM^−1^)	*K*_m_ (mM)	*k*_cat_ (s^−1^)	*k*_cat_/*K*_m_ (s^−1^mM^−1^)	*K*_m_ (mM)	*k*_cat_ (s^−1^)	*k*_cat_/*K*_m_ (s^−1^mM^-1^)
**1a**	2.36	7.03	2.98	2.45	7.61	3.11	1.85	10.23	5.53
**2a**	4.52	15.54	3.44	3.98	15.94	4.01	1.40	20.64	14.74
**3a**	4.33	5.36	1.24	4.12	6.35	1.54	2.39	9.34	3.91
**4a**	3.86	7.83	2.03	3.61	7.89	2.19	3.13	10.38	3.32
**5a**	3.10	8.36	2.70	3.23	9.13	2.83	1.84	13.64	7.41
**6a**	—	—	—	—	—	—	—	—	—
**7a**	4.86	7.20	1.48	4.73	7.81	1.65	4.51	9.37	2.08
**8a**	4.91	7.94	1.62	4.82	7.88	1.63	4.11	9.82	2.39
**9a**	4.73	7.90	1.67	4.50	8.11	1.80	1.98	10.14	5.12
**10a**	4.01	14.34	3.58	4.10	13.94	3.40	3.82	20.67	5.41
**11a**	3.17	15.24	4.81	3.12	16.72	5.36	1.86	23.41	12.59
**12a**	3.51	18.62	5.30	3.50	19.18	5.48	1.14	26.83	23.54
**13a**	—	—	—	—	—	—	—	—	—
**14a**	5.03	4.62	0.92	4.98	4.71	0.95	4.08	9.43	2.31

Each value was calculated depending on three independent measurements and all standard errors of fits were not more than 5%.

These improvements in the relative catalytic efficiency of crosslinked SCRII were attributable to stronger binding between enzyme and substrate. Crosslinked SCRII tended to show lower *K*_m_ values which probably associated with increased catalytic efficiency. For SCRII, crosslinking may have changed the intermolecular conformation or molecular interaction, resulting in the lower observed *K*_m_ values. Moreover, the possible cyclisation of crosslinked SCRII may help reduce conformational freedom and create constrained structural frameworks which often confer high receptor binding affinity and specificity^27, 28^.

### SrtA-mediated ligation of SDRs remarkably increases transformation efficiency with improved thermostability

The sortase-mediated method newly developed here was extended to the crosslinking of another eight SDRs (Table 1) for efficient chiral synthesis. The oxidoreductases ADHR, C1, C2, CR2, CR4, S1, SCR1 and SCR3 were selected to construct corresponding crosslinked oxidoreductases using SrtA-mediated method. The GGGGSLPETGG tag was added to their C-terminus. Unlike WT SCRII, these eight oxidoreductases did not have a native glycine at the N-terminus. Thus, a GGG tag was added to N-terminus of oxidoreductases’ genes. After overexpression, the eight oxidoreductases were successfully prepared with estimated purity >95% (Supplementary Fig. S4). Superdex 200 size exclusion chromatography revealed that ADHR, S1, SCR1 and SCR3 were tetrameric protein while C1, C2 and CR2 existed as monomeric form. The purified CR4 showed a subunit molecular mass of 32 kDa on SDS-PAGE but with an estimated native enzyme molecule weight of around 85 kDa by size exclusion chromatography. These results were consistent with previous reports^29–35^.

**Figure 7:**
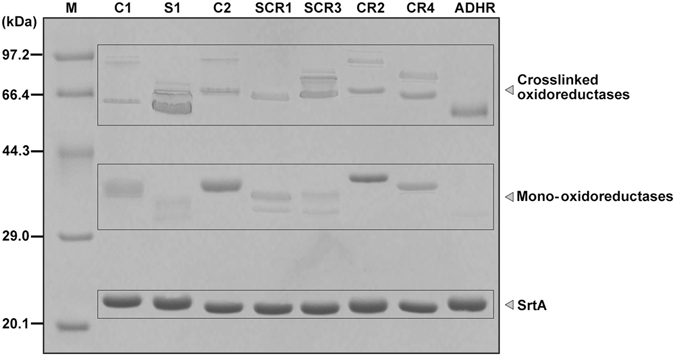
SDS-PAGE analysis of SrtA-mediated ligation of 8 oxidoreductases after incubating for 36 h. Mono-oxidoreductases included oxidoreductase-mtf, oxidoreductase-GGGGSLPET and cyclized oxidoreductase-GGGGSLPET. Ligation products, mainly dimers and trimers, were labelled as crosslinked oxidoreductases.

**Figure 8:**
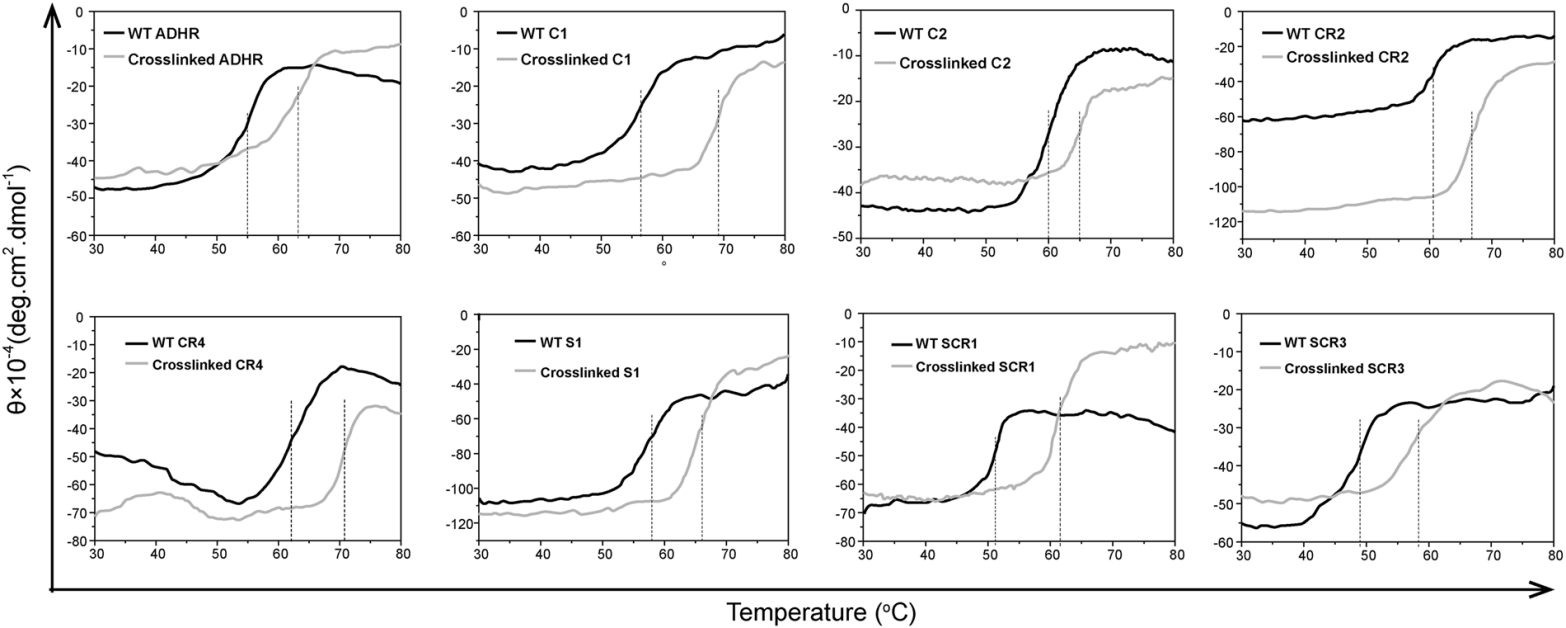
Thermal shift curves of 8 oxidoreductases and their crosslinked oxidoreductases. The buffer (5 mM phosphate buffer pH7.0, 0.1 mg/mL enzymes.) showed pH shift with no more than 0.5 while it was heated from 20 °C to 100 °C.

Based on the optimised conditions for SCRII ligation, the eight oxidoreductases were incubated with SrtA for 36 h at 25 °C. As shown in Fig. 7, all eight enzymes formed crosslinked oxidoreductases. Judged by molecular weights, most of the products were dimers and trimers while only dimers were observed for SCR1 and ADHR. MALDI-TOF-MS results also confirmed the ligation products (Supplementary Figs S5–12) Meanwhile, similar with SrtA-mediated crosslinking of SCRII, S1, SCR1 and SCR3 formed byproducts supposed to be cyclised monomers which were observed in SDS-PAGE below the position of the respective oxidoreductase-mtf. C1, C2, CR2, CR4 and ADHR formed almost no supposed cyclised monomers. Different distances between N-and C-termini in different proteins probably resulted in the different cyclisation behaviour. It is worth mentioning that monomeric ADHR was almost completely consumed after 36 h, suggesting that crosslinked ADHR might show similar steric hindrance of SrtA to that we proposed for crosslinked SCRII. The crosslinked oxidoreductases generated were separated from residual mono-oxidoreductases and SrtA by size exclusion chromatography. The crosslinked ADHR, CR4, S1, SCR1 and SCR3 may exist as tetramer and hexamer like crosslinked SCRII (Supplementary Figs S13–17). However, crosslinked C1, C2 and CR2 displayed no further oligomerisation according to the estimated molecular mass in size exclusion chromatography (Supplementary Figs S18–20). Perhaps the different quaternary structure of native enzymes caused different result. The denaturation temperatures of the 8 crosslinked oxidoreductases were measured by thermal shift assays. They were much more stable than their corresponding WT enzymes, with T_m_ values increased by 5–12 °C (Fig. 8). For example, the T_m_ value of crosslinked C1 was 69 °C, 12 °C higher than that of WT C1. This increase in the thermal stability could be due to more covalent bonds between the monomers of crosslinked C1 enzyme than WT enzyme according to Xiang *et al*.^36^.

We further determined the stereoselective reaction efficiency of the eight oxidoreductases and corresponding crosslinked enzymes toward their preferred substrates. The SrtA-mediated crosslinking resulted in 5- to 11-fold increases in the specific activities of these enzymes (Table 3). For example, the activity of crosslinked S1 in the reduction of **11a** was 10.8-fold greater than that of WT S1. Besides, the biotransformation was carried out and the products were examined by HPLC. All crosslinked oxidoreductases and their WT enzymes catalyzed the biotransformation of chiral alcohols with a similar optical purity of over 99% except for CR2 and SCR3 toward **1a** and **2a**, respectively. The crosslinked oxidoreductases improved the yield of chiral products by 25–285% compared to WT enzymes. They did not show any changes in the stereoselectivity of the reduction reactions, but performed much more effectively than WT enzymes. Kim *et al*. suggested that ligation brought target proteins together to form complementary interfaces, promoting higher catalytic efficiency through substrate channelling, high local substrate and enzyme concentrations and compartmentalisation^37^. Aboye *et al*. pointed out that cyclisation helped stabilize protein fold by reducing the backbone entropy associated with the unfolded state of a protein^38^. Like crosslinked SCRII, we suspected that the 8 crosslinked oxidoreductases possibly existed as cyclic form to have their structural frameworks constrained for high receptor binding affinity, specificity and enhanced stability. The improved thermal stability and catalytic efficiency of crosslinked oxidoreductases should also possibly be associated with the cyclic intermolecular conformation.

**Table 3:** Specific activity and stereoselective efficiency of WT oxidoreductases and crosslinked oxidoreductases toward their favorite substrates.

Enzymes	S	WT oxidoreductases			Crosslinked oxidoreductases		
		Activity ^a^ (μmol/(min·mg))	Cf ^b^ ee (%)	Yd ^b^ (%)	Activity ^a^ (μmol/(min·mg))	Cf ^b^ ee (%)	Yd^b^ (%)
ADHR	**10a**	1.04	*S*(>99)	27	6.82	*S*(>99)	67
C1	**13a**	0.86	*R*(>99)	23	6.11	*R*(>99)	61
C2	**13a**	2.12	*R*(>99)	43	11.32	*R*(>99)	>99
CR2	**1a**	0.93	*R*(81)	25	5.86	*R* (83)	65
CR4	**1a**	5.81	*R*(>99)	79	36.81	*R*(>99)	>99
S1	**11a**	0.29	*S*(>99)	13	3.14	*S*(>99)	50
SCR1	**2a**	1.05	*S*(>99)	30	8.31	*S*(>99)	97
SCR3	**2a**	2.11	*S*(98)	46	14.20	*S*(99)	99

^a^Standard assay conditions: 100 mM phosphate buffer (pH 7.0), 0.5 mM NADPH, 5 mM substrate, 1 mg/mL enzymes at 35 °C. The data was the representative of three independent experiments and standard errors were not more than 5%. ^b^The biotransformation was conducted with 5 g/L substrate and sufficient NADPH for 6 h.

## Conclusion

In summary, we have developed SrtA-mediated crosslinked short-chain dehydrogenases/reductases as novel biocatalysts with improved thermostability for efficient stereoselective reactions. To the best of our knowledge, this work first describes a generic technology platform for the preparation of stable biocatalysts for efficient chiral synthesis, which will be of industrial interest.

## Methods

### Chemicals

2-Hydroxyacetophenone derivatives including 3a, 4a, 5a, 6a were synthesized using the method described by Itsuno^39^. The following chemicals were purchased from Sigma-Aldrich (St. Louis, USA):, 2-hydroxyacetophenone 2a (98%), 2′-methylacetophenone 7a (98%), 3′-methylacetophenone 8a (98%), 2′-chloroacetophenone 10a (97%), 3′-chloroacetophenone 11a (98%), 4′-bromoacetophenone 12a (98%), 4′-methoxyacetophenone 13a (99%, Aldrich), butyrophenone 14a (≥99%), valerophenone 15a (99%), (*R*)-1-phenylethanol (99%), (*S*)-1-phenylethanol (98%), (*R*)-1-phenyl-1,2-ethanediol (99%), (*S*)-1-phenyl-1,2-ethanediol (99%) and (*S*)-1-(2-chlorophenyl)-1,2-ethanediol (96%). Acetophenone 1a (98%), 4′-methylacetophenone 9a (99%), NADPH and NADP+ (95%) were purchased from Aladdin Co. (Shanghai, China). All other racemic alcohol standards were prepared by reduction of ketones with sodium borohydride. Hexane and isopropanol for high performance liquid chromatography (HPLC) were of chromatographic grade from Sigma-Aldrich (St. Louis, USA). All other chemicals used were of the highest grade and available commercially.

Plasmids and strains. *Staphylococcus aureus* ST541 was used as sortase A (SrtA) gene donor. Its genomic DNA was extracted using Rapid Bacterial Genomic DNA Isolation Kit (Sangon Biotech. Co., Shanghai, China). Two primers *srtA*_F (*Nde*I) and *srtA*_R (*Xho*I) were designed to amplify core sequences (178−612 bp) of sortase A gene (*srtA*)^17^. The PrimeSTAR^®^ HS (Premix) DNA polymerase and *Taq* DNA polymerase for PCR were purchased from TAKARA (Otsu, Japan). PCR details were shown in Supplementary Table S3. The fragment of *srtA* was inserted into the corresponding site of the plasmid pET-21a (+). The plasmid pET-SrtA was then transformed into *Escherichia coli* BL21 (DE3) and verified by DNA sequencing. The recombinant *E. coli* strains containing ADHR, C1, C2, CR2, CR4, S1, SCR1, SCRII, SCR3 were previously constructed in our lab^40^. The primers were listed in Supplementary Table S3 for the construction of ADHR-mtf, C1-mtf, C2-mtf, CR2-mtf, CR4-mtf, S1-mtf, SCR1-mtf, SCRII-mtf, SCR3-mtf with N-terminal (Gly)_n_ and C-terminal GGGGSLPETGG motif. The plasmid pET-28a (+) (Novagen, USA) and strain *E. coli* BL21 (DE3) were used for gene expression. Plasmid DNA was further sequenced to confirm the positive clones.

### Protein expression and purification

The protein expression and purification was carried out as described previously with minor modification^41^. The recombinant strains were grown in LB medium containing 100 μg/mL ampicillin (for *E. coli* BL21/pET-SrtA) or 50 μg/mL kanamycin (for other strains) at 37 °C. When *OD*_600_ value of culture reached 0.6–0.8, 0.1 mM isopropyl β-D-thiogalactoside (IPTG) was added to induce protein expression at 25 °C for 10 h. Buffer was titrated with corresponding acid/base. The pH meter (Mettler Toledo, Switzerland) was calibrated at 25 °C using standard buffer provided by manufacturer. The cells were harvested by centrifugation and suspended in buffer A (20 mM Tris-HCl, 150 mM NaCl, pH 8.0), and subsequently lysed by sonication at 4 °C. The supernatant was collected and applied to a HisTrap HP affinity column (GE Healthcare, Piscataway, NJ, USA) equilibrated with buffer A. With buffer B (20 mM Tris-HCl, 150 mM NaCl, 1 M imidazole, pH8.0), linear elution was carried out using an AKTA purifier system (GE Healthcare, Piscataway, NJ, USA). Then, the pooled fractions were further loaded on a Resource Q column (1 by 1 cm) equilibrated with the buffer (20 mM Tris-HCl, pH 8.0). Finally, the fractions were applied to a Superdex 200 HiLoad 26/60 (GE Healthcare, Piscataway, NJ, USA) for chromatography in a buffer containing 20 mM Tris-HCl and 150 mM NaCl (pH8.0). The homogeneity of purified enzymes was judged by Coomassie brilliant blue staining of sodium dodecyl sulfate-polyacrylamide gel electrophoresis (SDS-PAGE). Visualized protein bands were scanned by the FluorChem E imaging station (ProteinSimple, USA). Relative intensity of each band was calculated with the software Bandscan 5.0 to estimate the content of targeted protein.

### SrtA mediated ligation of oxidoreductases

For the SrtA-mediated ligation of SCRII, the reaction mixture containing 35 μM SCRII-mtf and 20 μM SrtA was incubated in a ligation buffer (50 mM Tris-HCl, 150 mM NaCl, 10 mM CaCl_2_, pH 7.5) at 10 °C, 15 °C, 20 °C, 25 °C, 30 °C and 35 °C, respectively for 8 h. The ligation samples were taken for SDS-PAGE analysis. According to the calculated relative intensity of each band using Band 5.0, the lane with the most products was corresponding to the optimal temperature. Afterwards, the SrtA-mediated ligation of SCRII-mtf and other 8 oxidoreductases were conducted under the optimal condition for 36 h.

### Purification of crosslinked oxidoreductases

The SrtA-mediated ligation mixture was concentrated and desalted with an AMICON Ultra-15 (MWCO 10 kDa, Millipore, Bedford, USA). The desalted samples were subjected to a Superdex 200 10/300 GL column (GE Healthcare, Piscataway, NJ, USA) equilibrated with a buffer containing 50 mM Tris-HCl and 150 mM NaCl (pH 8.0). The eluted fractions were collected for SDS-PAGE analysis and the crosslinked oxidoreductases and residual monomers of oxidoreductases and SrtA were separated.

### MALDI-TOF-MS analysis of ligation product

The bands below SCRII-mtf on SDS-PAGE gel were excised for MALDI-TOF-MS analysis with Bruker Daltonics FLEX (Billerica, USA), followed by peptide mass fingerprinting analysis with Proteomics solution I system. The peptide mass data were used to query the Mascot database (http://www.matrixscience.com). The purified crosslinked oxidoreductases were dissolved in distilled water (10 mg/ml). 2,5-Dihydroxybenzoic acid was prepared as 1% (w/v) solution and used as a matrix. Then the sample solution and matrix were mixed in equal volumes (1.0 μl each), spotted on a sample plate followed by air-drying until homogeneous crystals formed. The dried sample on the plate was then applied to the mass spectrometer and analyzed with an accelerated voltage and reflective voltage of 19 kV and 16 kV, respectively.

### Enzyme assay and kinetic determination

The enzyme assay mixture in 250 μL comprised 100 mM potassium phosphate buffer (pH 6.0), 0.5 mM NADPH, 5 mM substrate, and enzymes (1 mg/mL). The reductive activity on substrates was measured at 20–70 °C by recording the rate of change in NADPH absorbance at 340 nm. One unit of enzyme activity is defined as the amount of enzyme catalyzing the oxidation of 1 μmol of NADPH per minute under measurement condition. The relative activity was expressed as percentage of maximum activity under experimental conditions.

The kinetic parameters of WT SCRII, SCRII-mtf and crosslinked SCRII were measured and calculated using a Beckman DU-7500 spectrophotometer with a Multicomponent/SCA/Kinetics Plus software package and a thermostated circulating water bath. Substrate (0.5 to 20 mM), enzyme (~30 μM, 1 mg/mL), and cofactor NADPH (5 mM) in 100 mM potassium phosphate buffer (pH 6.0) were used for a series of assays. Each value was calculated depending on three independent measurements and all standard errors of fits were not more than 5%. The data obtained were fitted to the function v = V_max_[s]/(*K*_m_ + [s]). Kinetic parameters were derived from Michaelis-Menten plots and Lineweaver-Burk.

### Biotransformation and analytical methods

The biotransformation was carried out as described previously with minor modification^42^. The reaction mixture in a 2-mL volume consisted of 100 mM potassium phosphate buffer (pH 6.0/7.0), 5 g/L substrate, sufficient NADPH, and pure enzymes (1 mg/mL). The reactions were carried out at 35 °C for 6 h with shaking at 150 rpm respectively. Time sampling was performed every 30 min if necessary. The product was extracted with ethyl acetate, and the organic layer was used for analysis. The optical purity and yield of product were determined by high-performance liquid chromatography on a Chiralcel OB-H column (Daicel Chemical Ind. Ltd., Japan). Method details including flow rates, mobile phase and retention time of products are shown in Supplementary Figs S21–S32.

### Circular dichroism spectroscopy and thermal shift assay

Circular dichroism (CD) measurements were performed with a Jasco J720 spectropolarimeter (Jasco, Inc., Easton, USA). Data of absorbed wavelength from 190 to 250 nm were collected within a phosphate buffer (pH 6.0) using the following instrument settings (for an average of 30 scans): response, 1 s; sensitivity, 100 mdeg; speed, 50 nm/min, average of 30 scans. The protein concentration was ~3 μM (~0.1 mg/mL) in 100 mM potassium phosphate buffer (pH 6.0). The CD data was further deconvolved by K2d method (Dichroweb server) to determine the content of secondary structures. The thermal denaturation was carried out by observing the impact of temperature on protein secondary structure. The protein samples were heated from 20 °C to 90 °C and then cooled to 20 °C by a Jasco programmable Peltier element with a heating/cooling rate of 1 °C/min. The wavelength of 209 nm that characterized the α-helix within protein was used to monitor the unfolding rate of the protein structures. The proteins were diluted to 0.1 mg/mL within 5 mM potassium phosphate buffer (pH 6.0/7.0) which had been conducted for a heating study to observe pH shift. The solvent contribution was discounted to correct the obtained spectra. The denaturation temperature (T_m_) was calculated according to the maximum slope that signified the swift unfolding of secondary structures.

## Electronic supplementary material

Supplementary information
