# Supplementary material for: Sortase A-mediated crosslinked short-chain dehydrogenases/reductases as novel biocatalysts with improved thermostability and catalytic efficiency
Source: Sci Rep. 2017 Jun 8;7:3081. doi: 10.1038/s41598-017-03168-z (PMC5465079; doi:10.1038/s41598-017-03168-z)
Supplement: Supplementary file 1 — Supplementary information [file 41598_2017_3168_MOESM1_ESM.pdf]

Supplementary information

Sortase A-mediated crosslinked short-chain dehydrogenases/reductases as novel biocatalysts with improved thermostability and catalytic efficiency

Kunpeng Li[a], Rongzhen Zhang[a, b*], Yan Xu[a, b*], Zhimeng Wu[a], Jing Li[a], Xiaotian Zhou[a], Jiawei Jiang[a], Haiyan Liu[a], Rong Xiao[c]

[a] Key Laboratory of Industrial Biotechnology of Ministry of Education & School of Biotechnology, Jiangnan University, Wuxi 214122, P. R.China

[b] National Key Laboratory for Food Science, Jiangnan University, Wuxi 214122, P. R. China

[c] Center for Advanced Biotechnology and Medicine, Rutgers University, Piscataway, NJ 08854, USA

***Corresponding author:** Rongzhen Zhang and Yan Xu

Tel: +86-510-85918201**; Fax:** +86-510-85864112

Email address: rzzhang@jiangnan.edu.cn, yxu@jiangnan.edu.cn

Present address: School of Biotechnology, Jiangnan University, 1800 Lihu Avenue, Wuxi, 214122, P. R. China

Supplementary Figures

Figure S1. Expression and purification of sortase A.

Figure S2. Mascot search results of peptide mass figureprint (PMF) to identify the bands below SCRII-mtf.

Figure S3. Biotransformation time determination for WT-SCRII, SCRII-mtf and crosslinked SCRII.

Figure S4. Purification of SCRII and other eight SDRs.

Figure S5-S12. MALDI-TOF-MS analysis of the ligation product of ADHR, C1, C2, CR2, CR4, S1, SCR1 and SCR3.

Figure S13-S20. Purification of of crosslinked ADHR, C1, C2, CR2, CR4, S1, SCR1 and SCR3 by size exclusion chromatography.

Figure S21-S32. HPLC analysis for determination of enantiomeric excess of alcohol products.

Supplementary Tables

Table S1. Secondary structure contents (%) of WT SCRII, SCRII-mtf and crosslinked SCRII.

Table S2 Michaelis-Menten and Lineweaver-Burk plots of WT SCRII, SCRII-mtf and crosslinked SCRII towards aryl ketone substrates.

Table S3. Primers in this work and PCR thermal cycle detail.

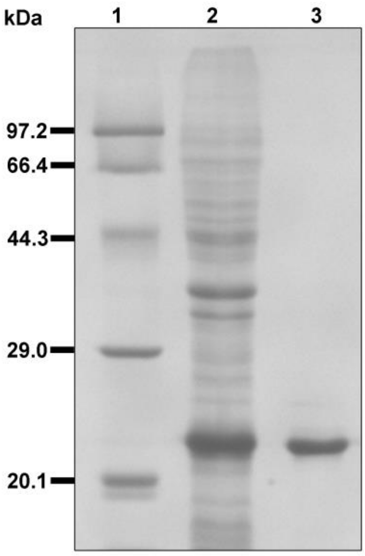

Figure S1. Expression and purification of sortase A. Lane 1, protein molecular weight marker (Low); Lane 2, the cell-free extracts of the recombinant *E. coli* BL21/pET-SrtA, the yield of SrtA is about 50 mg/l culture; Lane3, The purified SrtA (>90% purity).

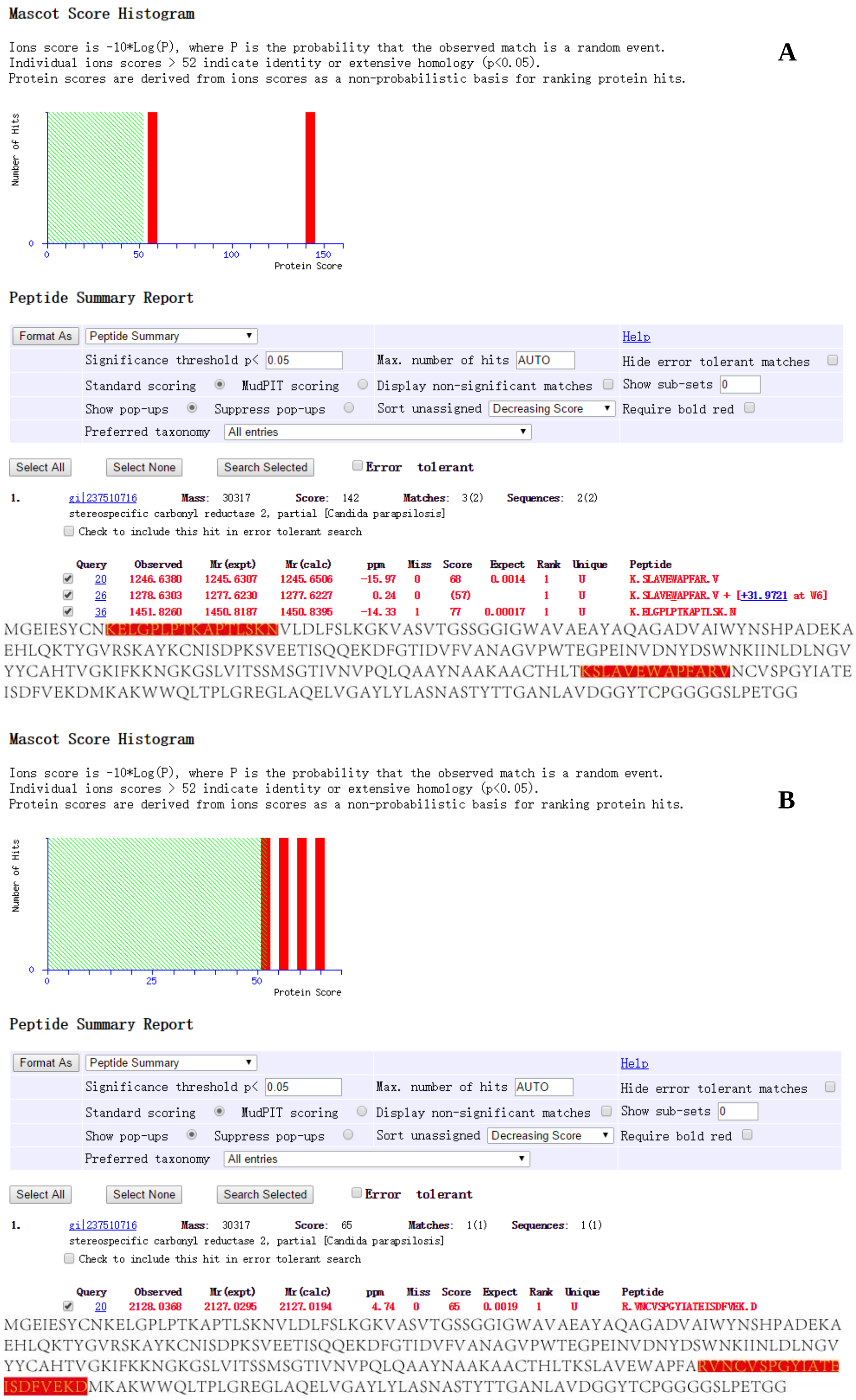

Figure S2. Mascot search results of peptide mass fingerprint (PMF) to identify the bands below SCRII-mtf. The bands below SCRII-mtf on SDS-PAGE gel were excised for MALDI-TOF-MS analysis with Bruker Daltonics FLEX (Billerica, USA), followed by peptide mass fingerprinting analysis with Proteomics solution I system. The peptide mass data were used to query the Mascot database (http://www.matrixscience.com). The peptide sequences detected by PMF was indicated in the protein sequence of SCRII-mtf. Both the first band (A) and the second band (B) below SCRII-mtf were most like SCRII.

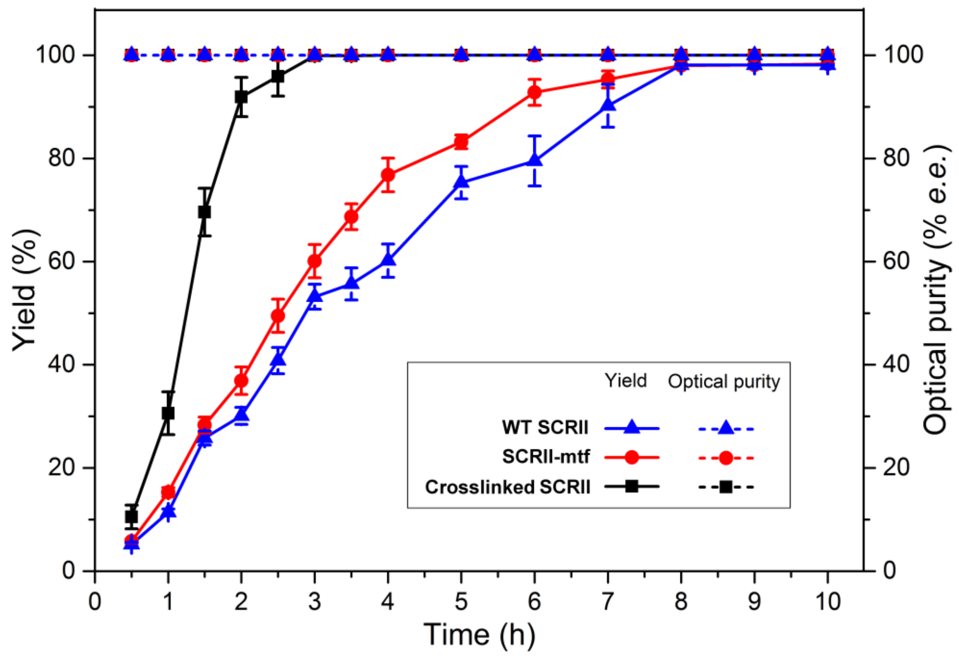

Figure S3. Biotransformation time determination for WT SCRII, SCRII-mtf and crosslinked SCRII. The reaction mixture in a 2-ml volume consisted of 100 mM potassium phosphate buffer (pH 6.0), 5 g/L substrate, sufficient NADPH, and an appropriate amount of pure enzymes (1mg/mL). The reactions were carried out at 35 °C with shaking at 150 rpm.

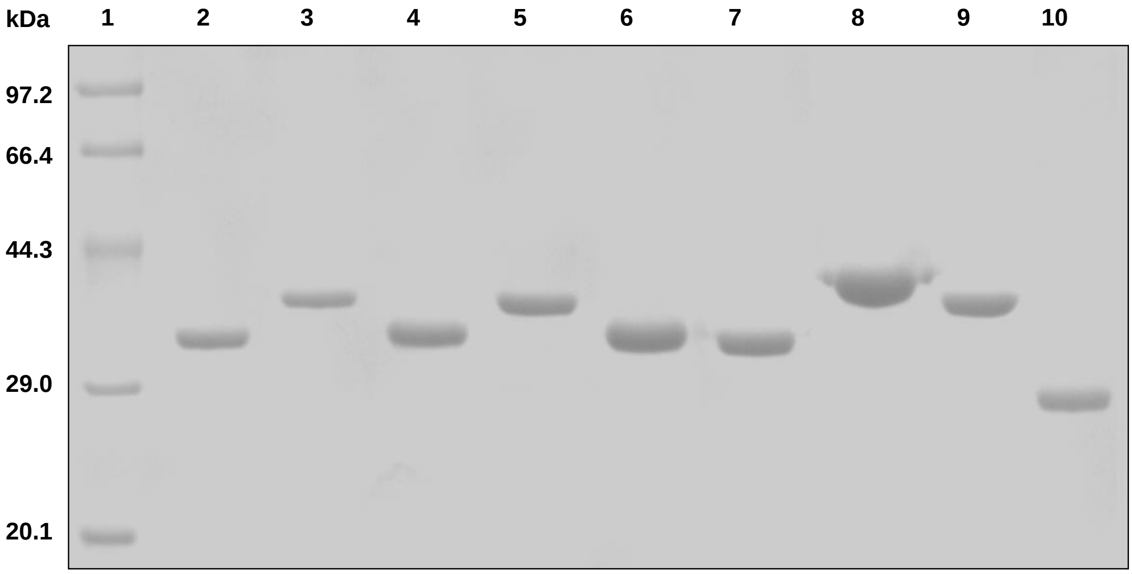

Figure S4. Purification of SCRII and other eight oxidoreductases. The purity of all 9 enzymes is above 95%. Lane 1, protein molecular weight marker (Low); Lane 2, SCRII-mtf; Lane 3, C1-mtf; S1-mtf; Lane 4, C2-mtf; Lane 5, SCR1-mtf; Lane 6, SCR3-mtf; Lane 7, CR2-mtf; Lane 8, CR4-mtf; Lane 9, ADHR-mtf.

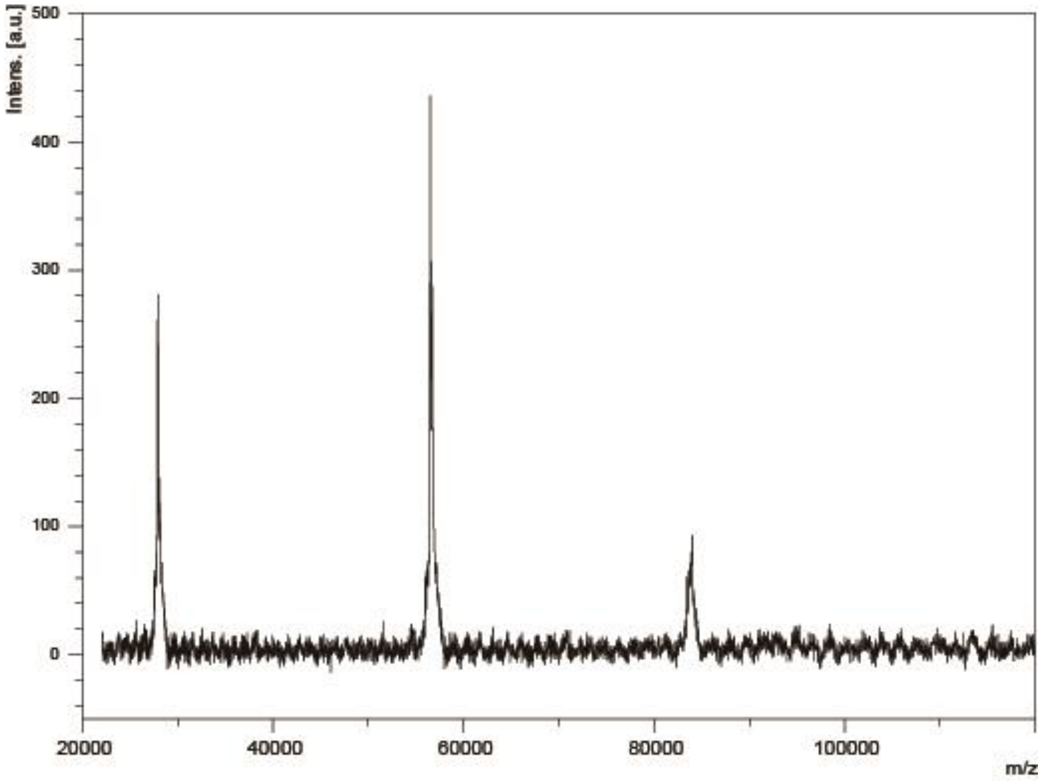

Figure S5. MALDI-TOF-MS analysis of the ligation product of ADHR.

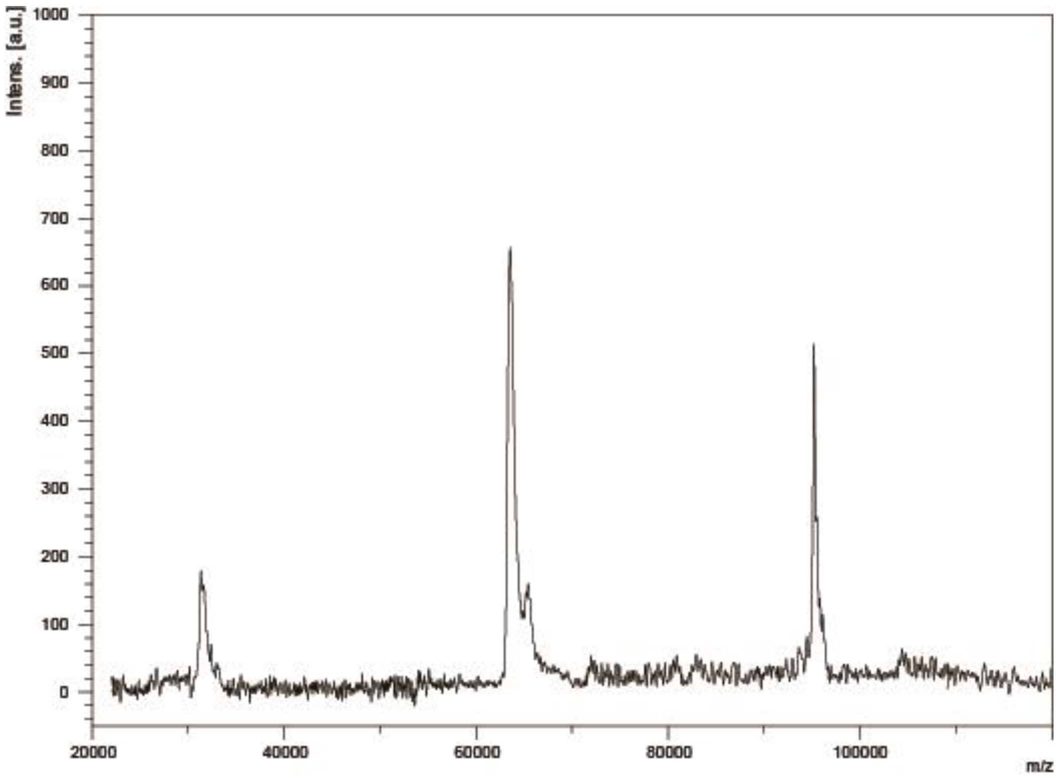

Figure S6. MALDI-TOF-MS analysis of the ligation product of C1.

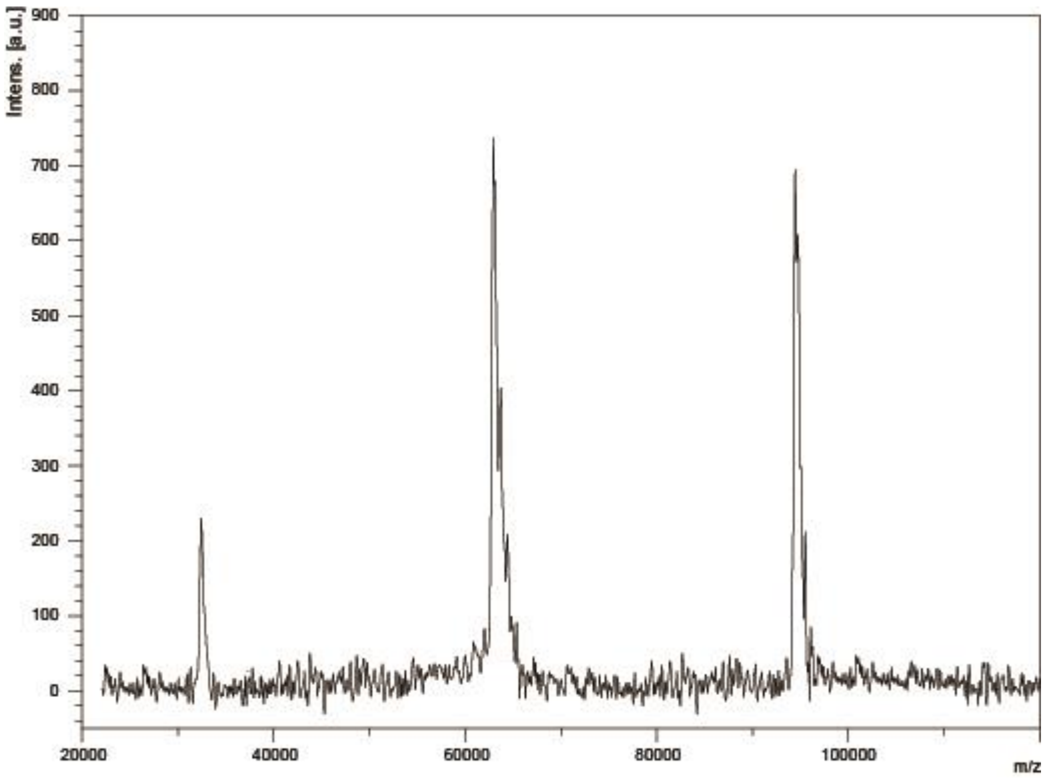

Figure S7. MALDI-TOF-MS analysis of the ligation product of C2.

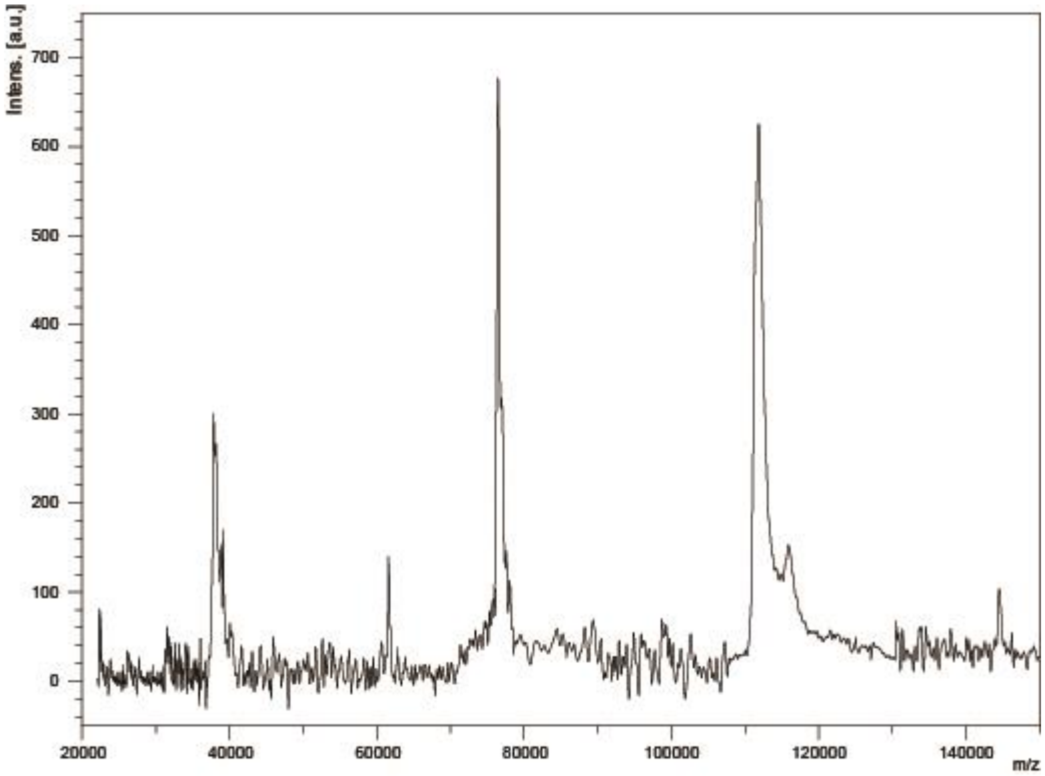

Figure S8. MALDI-TOF-MS analysis of the ligation product of CR2.

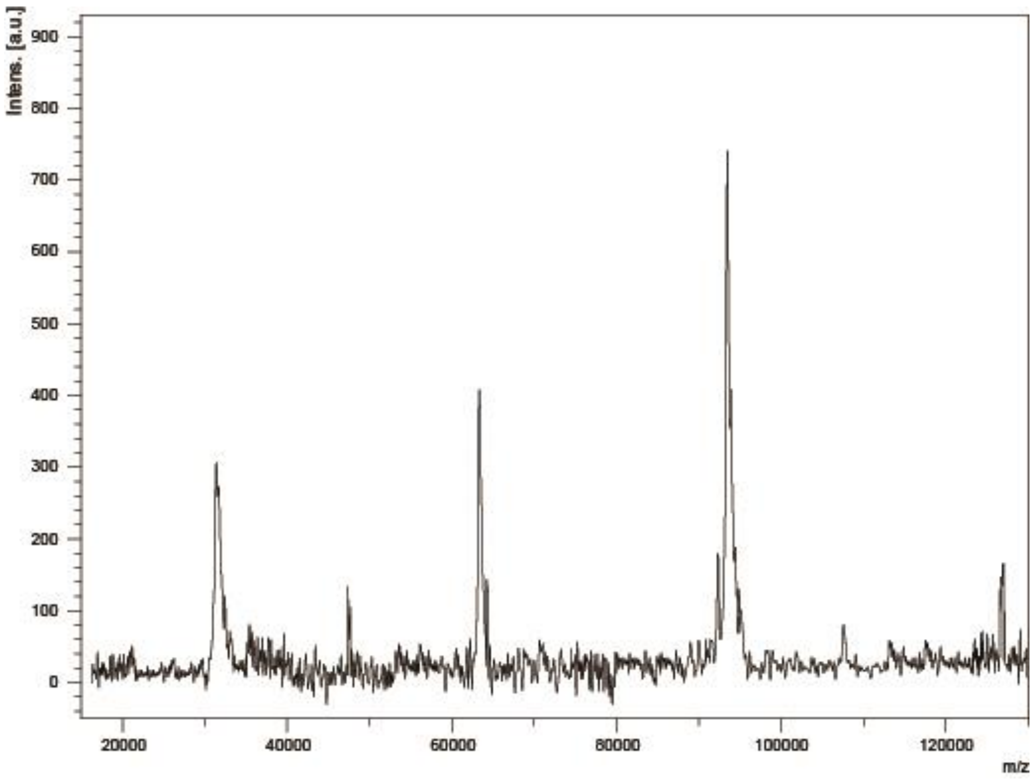

Figure S9. MALDI-TOF-MS analysis of the ligation product of CR4.

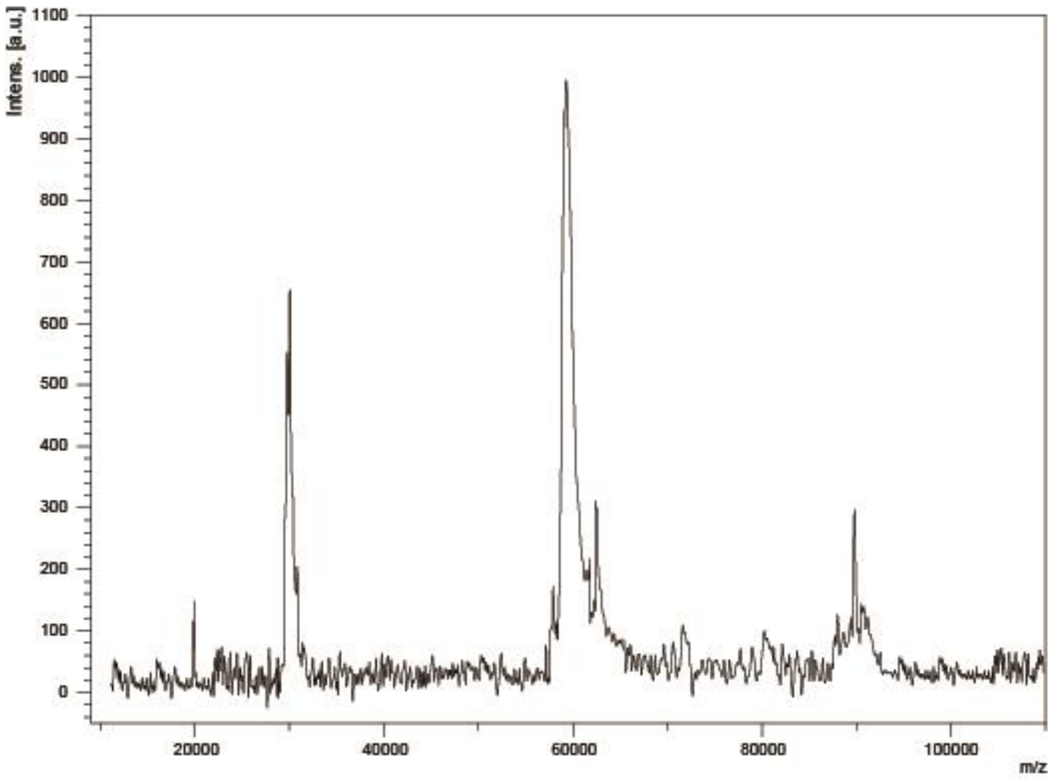

Figure S10. MALDI-TOF-MS analysis of the ligation product of S1.

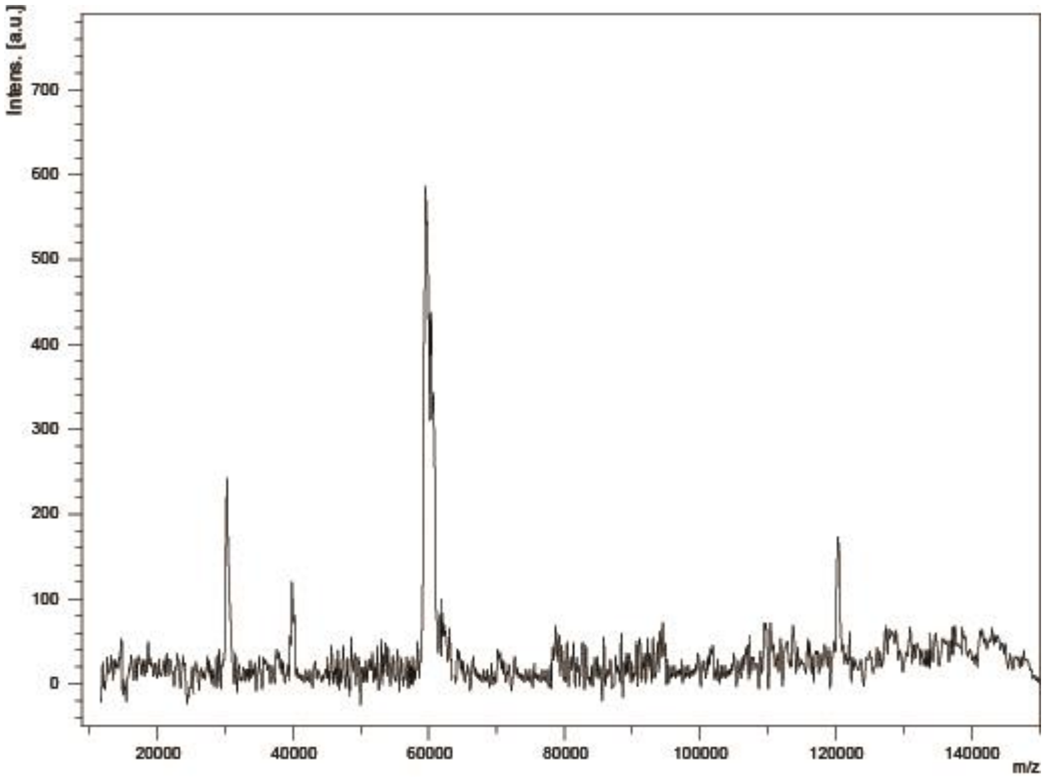

Figure S11. MALDI-TOF-MS analysis of the ligation product of SCR1.

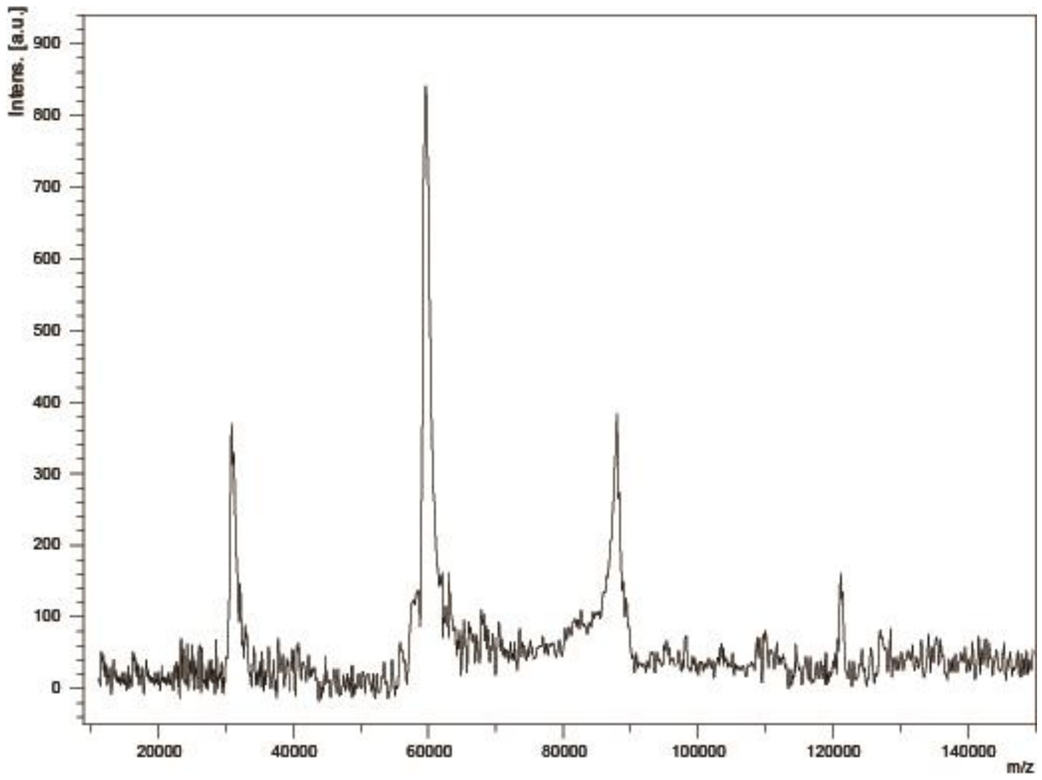

Figure S12. MALDI-TOF-MS analysis of the ligation product of SCR3.

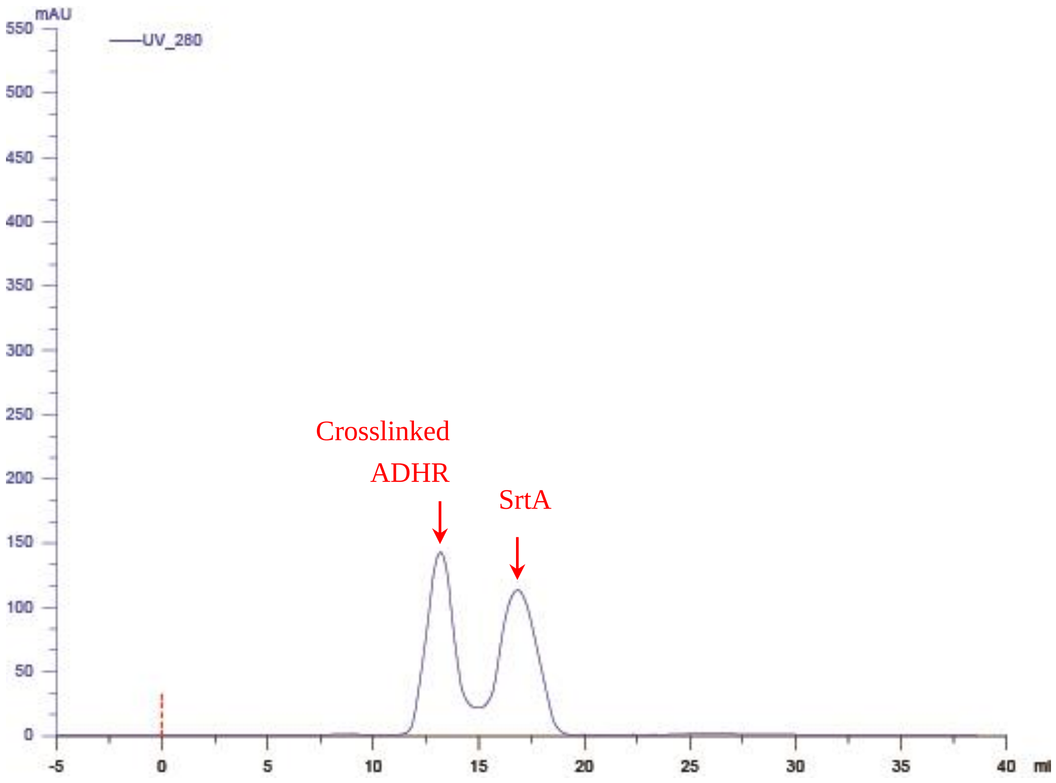

Figure S13. Purification of of crosslinked ADHR by size exclusion chromatography.

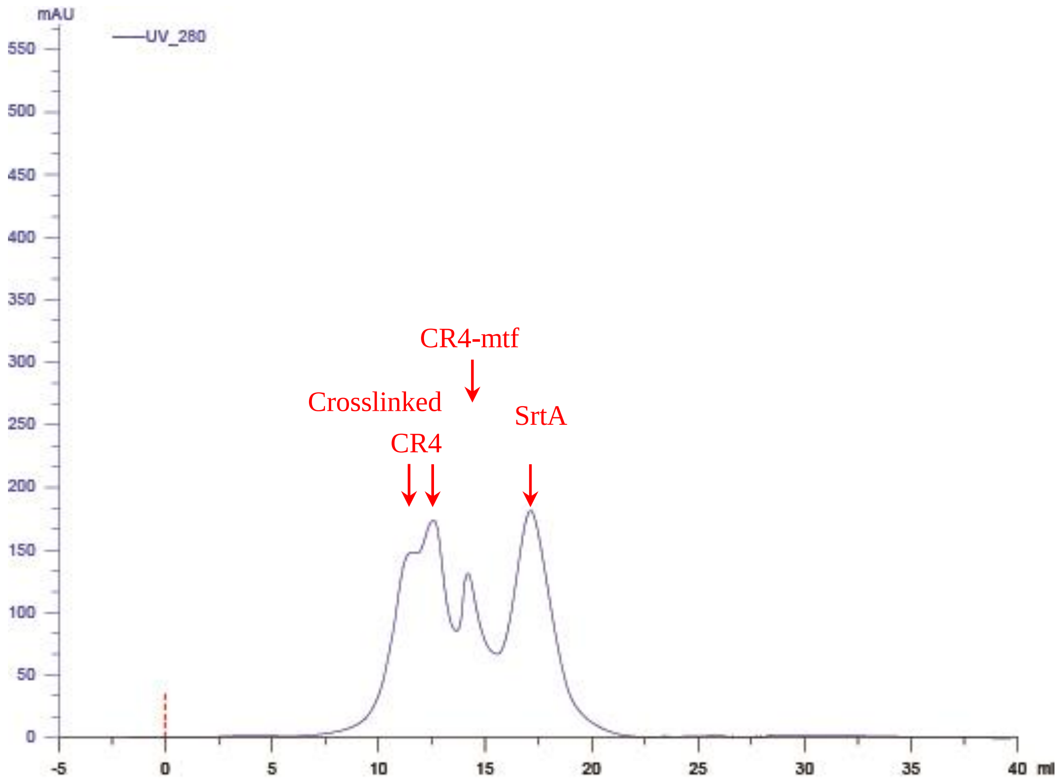

Figure S14. Purification of of crosslinked CR4 by size exclusion chromatography.

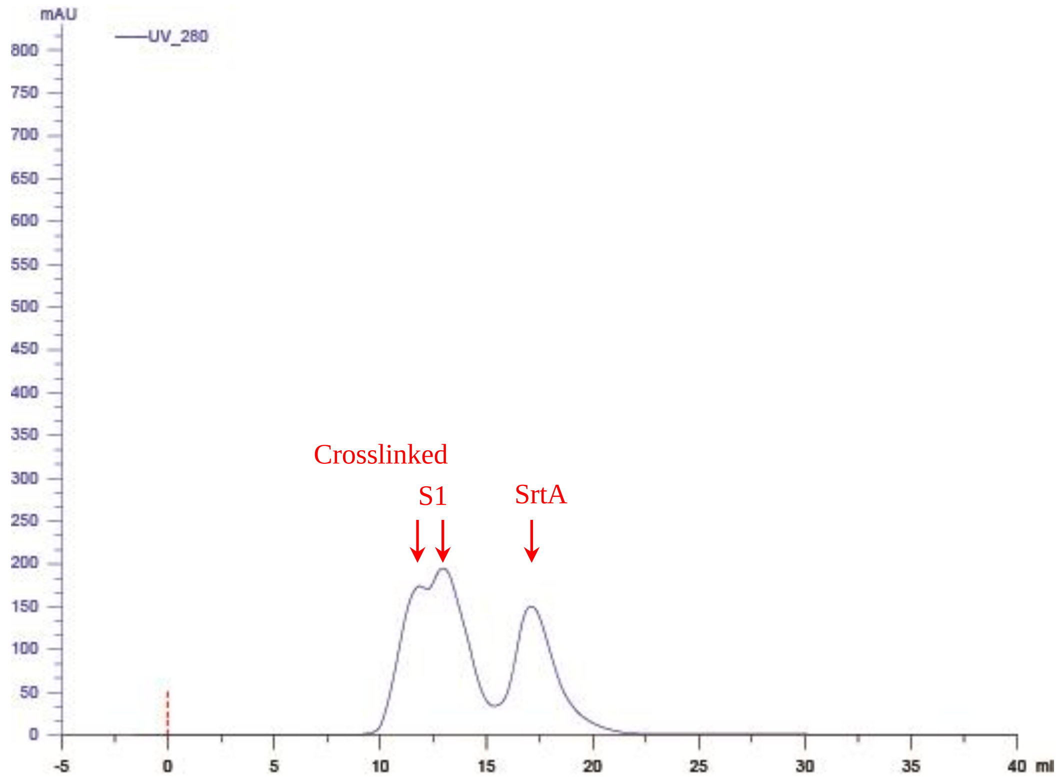

Figure S15. Purification of of crosslinked S1 by size exclusion chromatography.

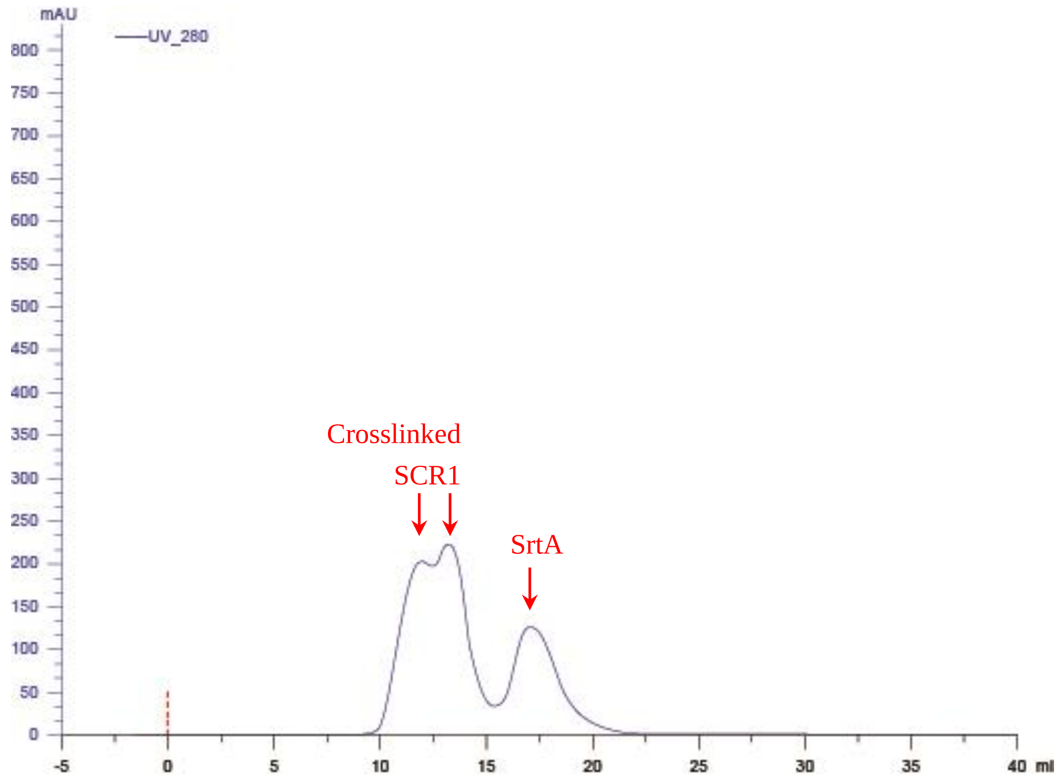

Figure S16. Purification of of crosslinked SCR1 by size exclusion chromatography.

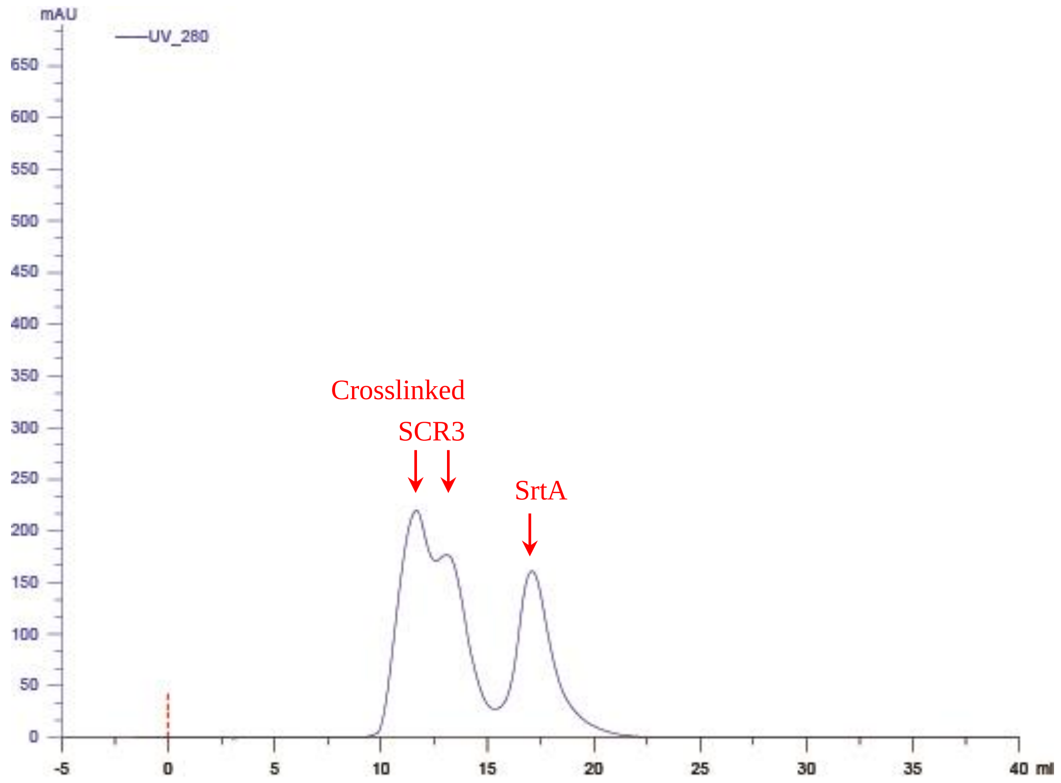

Figure S17. Purification of of crosslinked SCR3 by size exclusion chromatography.

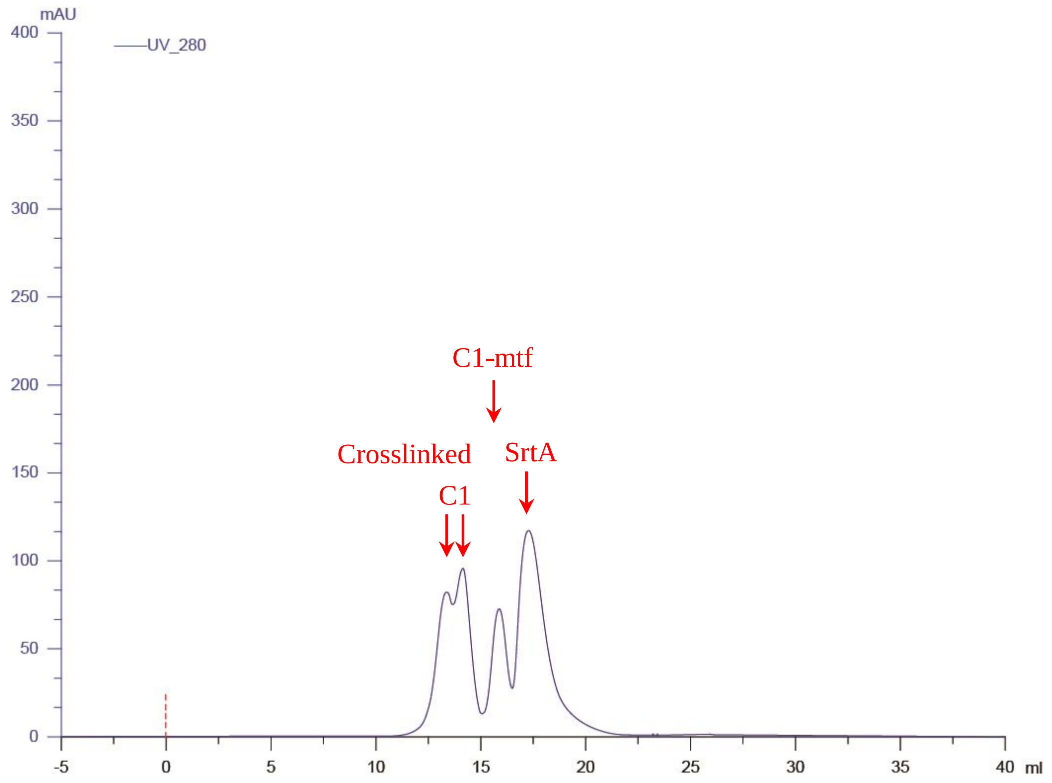

Figure S18. Purification of of crosslinked C1 by size exclusion chromatography.

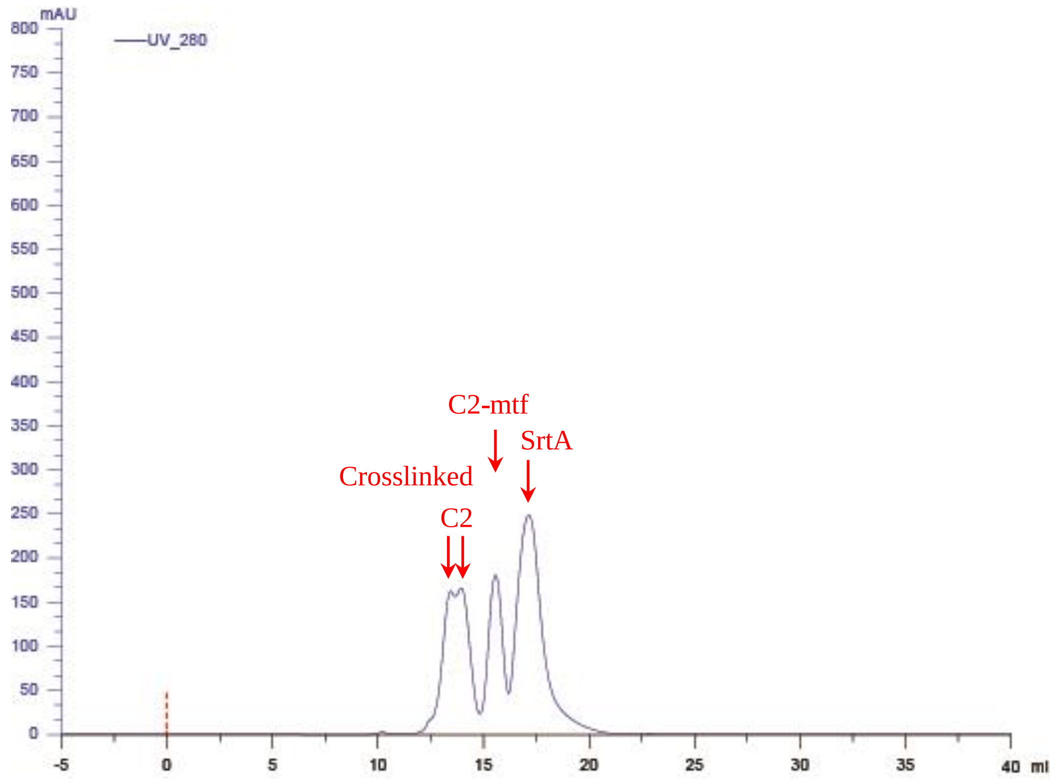

Figure S19. Purification of of crosslinked C2 by size exclusion chromatography.

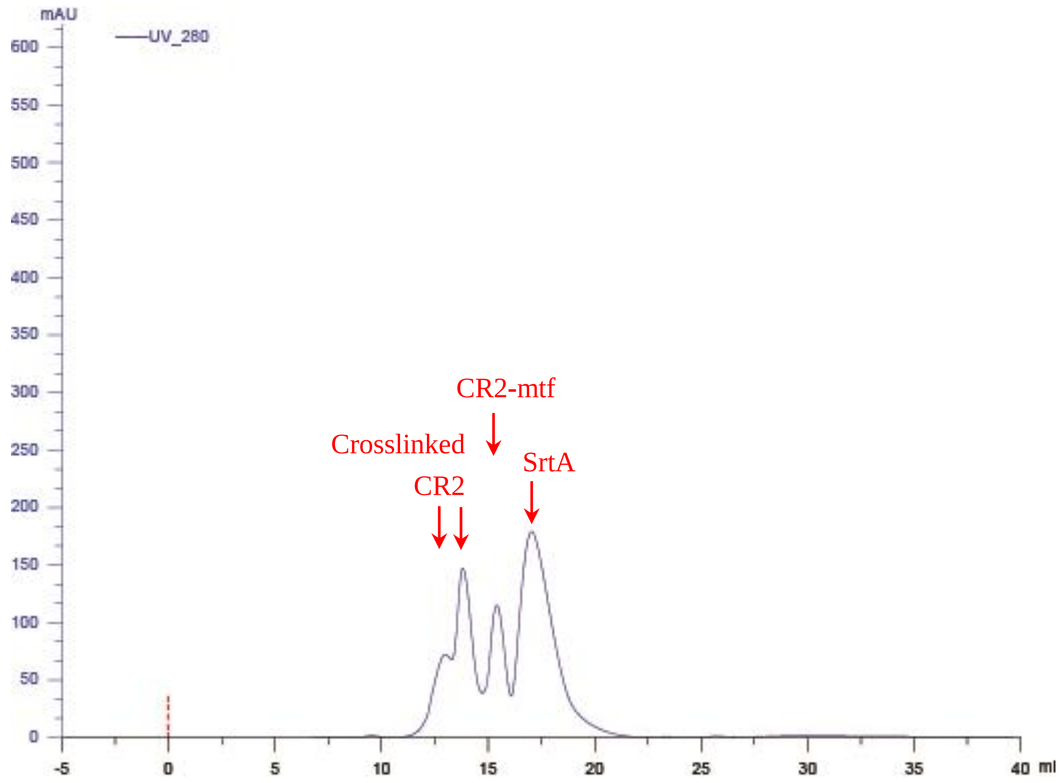

Figure S20. Purification of of crosslinked CR2 by size exclusion chromatography.

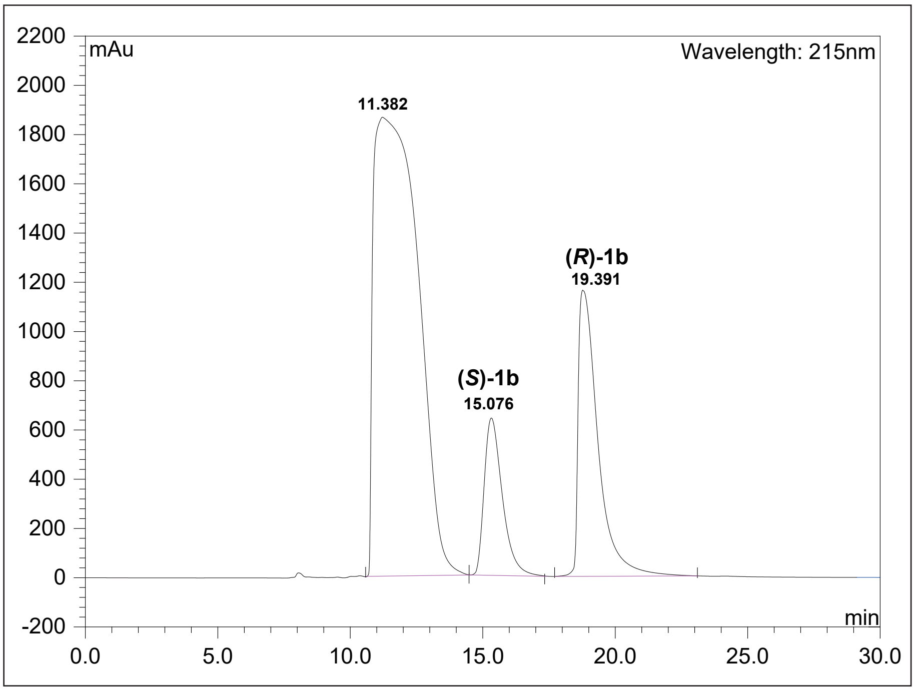

Figure S21. HPLC analysis for determination of enantiomeric excess of 1b: column OB-H; flow rate: 0.4 ml/min; Mobile phase hexane/isopropanol (***v***/***v***) = 9:1; (*R*)-1b = 19.4 min; (*S*)-1b = 15.1 min.

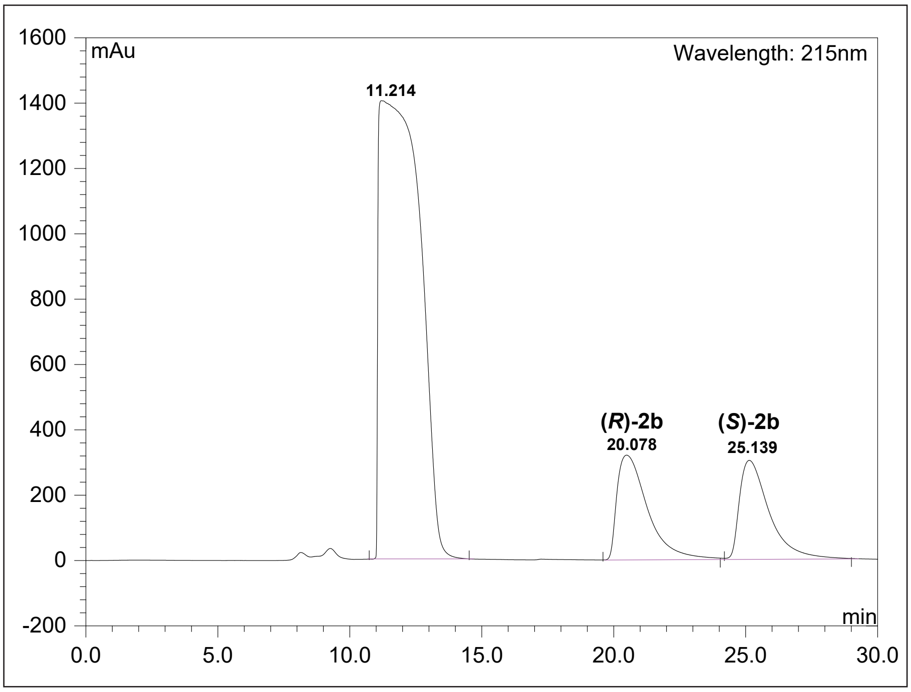

Figure S22. HPLC analysis for determination of enantiomeric excess of 2b: column OB-H; flow rate: 0.4 ml/min; Mobile phase hexane/isopropanol (***v***/***v***) = 9:1; (*R*)-2b = 20.1 min; (*S*)-2b = 25.1 min.

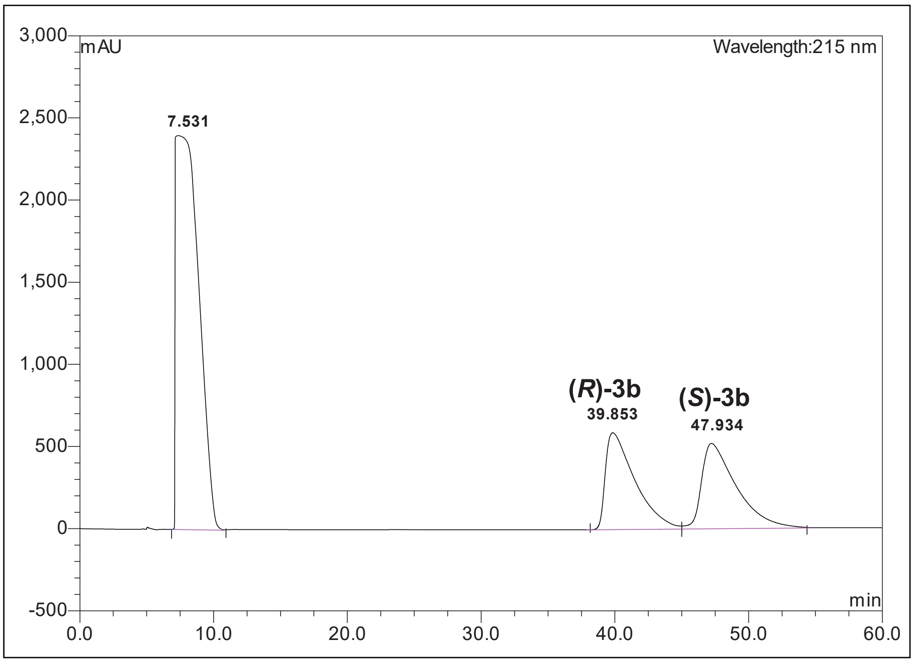

Figure S23. HPLC analysis for determination of enantiomeric excess of 3b: column OB-H; flow rate: 0.8 ml/min; Mobile phase hexane/isopropanol (***v***/***v***) = 98:2; (*R*)-3b = 39.9 min; (*S*)-3b = 47.9 min.

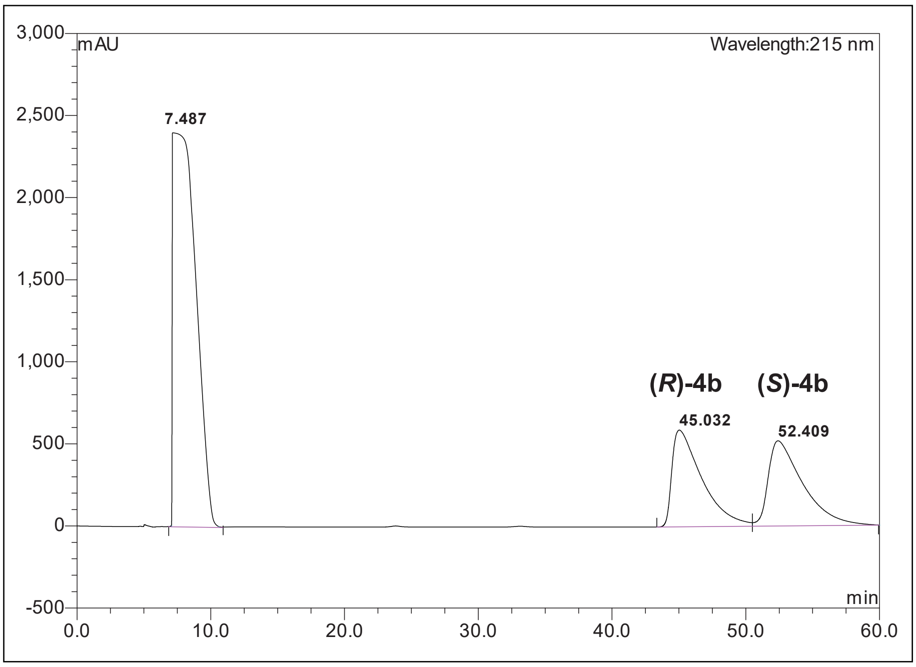

Figure S24. HPLC analysis for determination of enantiomeric excess of 4b: column OB-H; flow rate: 0.8 ml/min; Mobile phase hexane/isopropanol (***v***/***v***) = 98:2; (*R*)-4b = 45.0 min; (*S*)-4b = 52.4 min.

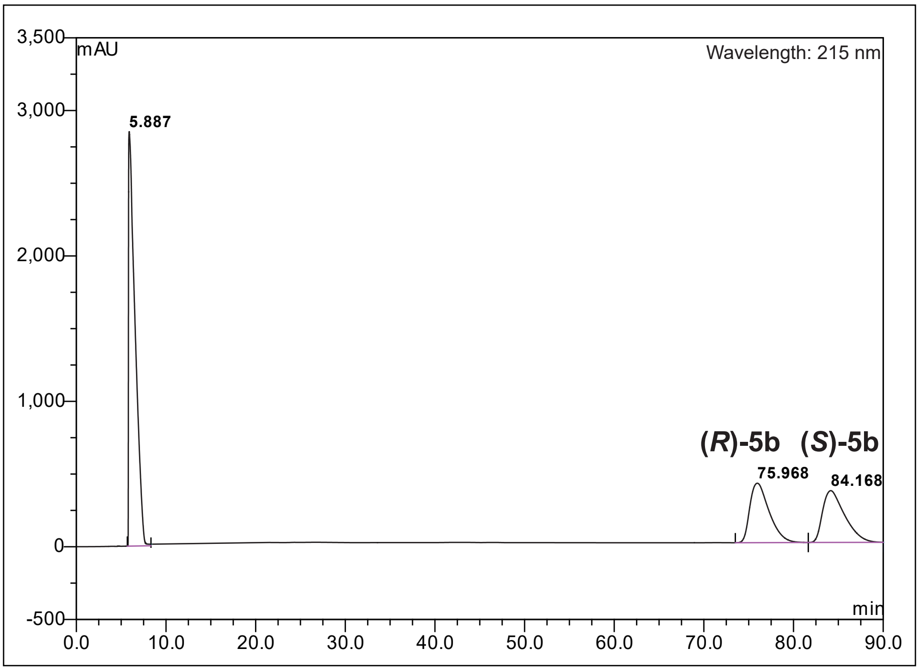

Figure S25. HPLC analysis for determination of enantiomeric excess of 5b: column OB-H; flow rate: 0.8 ml/min; Mobile phase hexane/isopropanol (***v***/***v***) = 98:2; (*R*)-5b = 76.0 min; (*S*)-5b = 84.2 min.

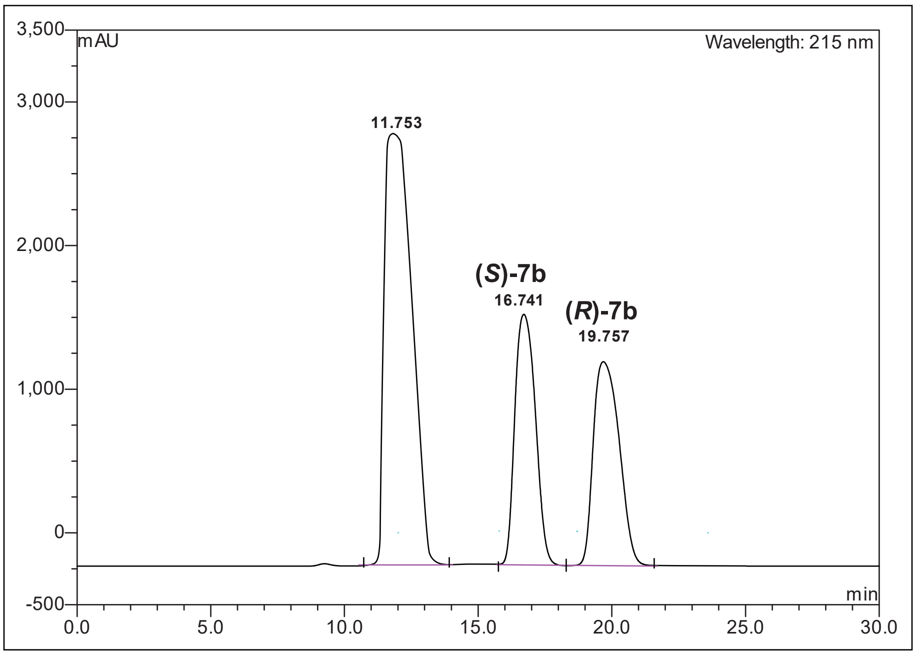

Figure S26. HPLC analysis for determination of enantiomeric excess of 7b: column OB-H; flow rate: 0.4 ml/min; Mobile phase hexane/isopropanol (***v***/***v***) = 9:1; (*R*)-7b = 19.8 min; (*S*)-7b =16.7 min.

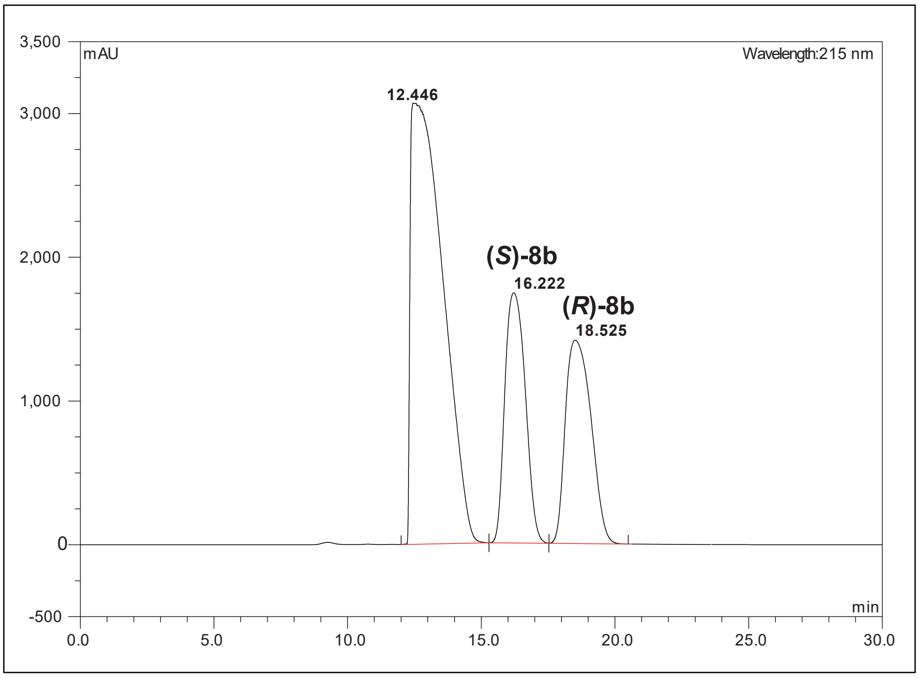

Figure S27. HPLC analysis for determination of enantiomeric excess of 8b: column OB-H; flow rate: 0.4 ml/min; Mobile phase hexane/isopropanol (***v***/***v***) = 9:1; (*R*)-8b = 18.5 min; (*S*)-8b = 16.2 min.

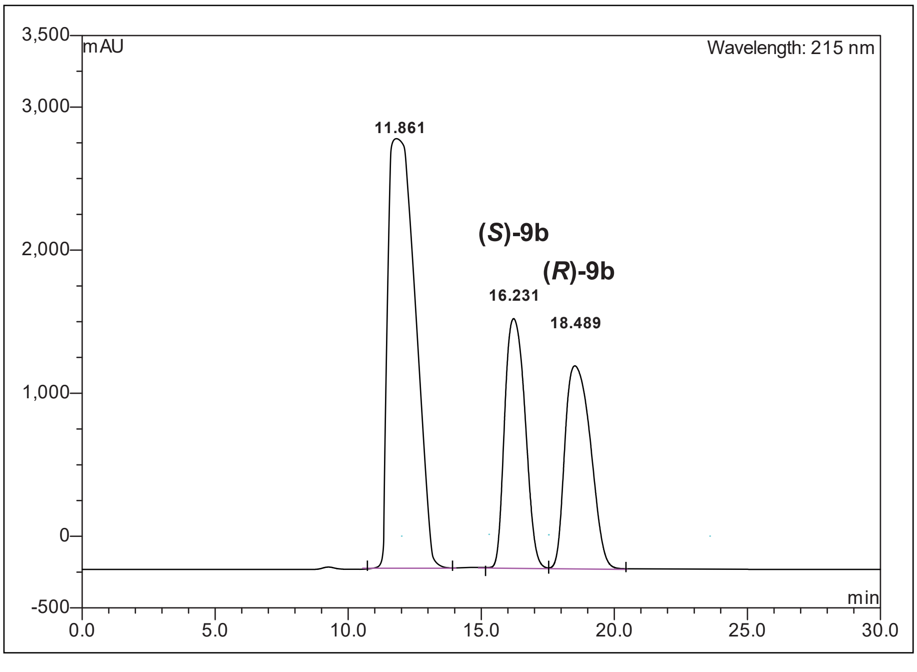

Figure S28. HPLC analysis for determination of enantiomeric excess of 7b: column OB-H; flow rate: 0.4 ml/min; Mobile phase hexane/isopropanol (***v***/***v***) = 9:1; (*R*)-9b = 18.5 min; (*S*)-9b =16.2 min.

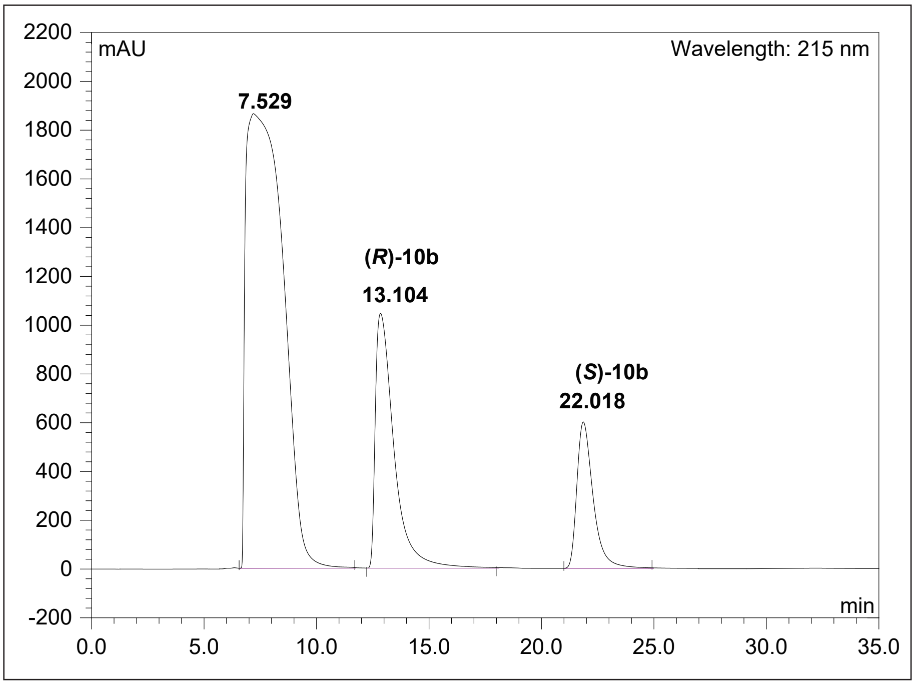

Figure S29. HPLC analysis for determination of enantiomeric excess of 10b: column OB-H; flow rate: 0.8 ml/min; Mobile phase hexane/isopropanol (***v***/***v***) = 98:2; (*R*)-10b = 13.1 min; (*S*)-10b = 22.0 min.

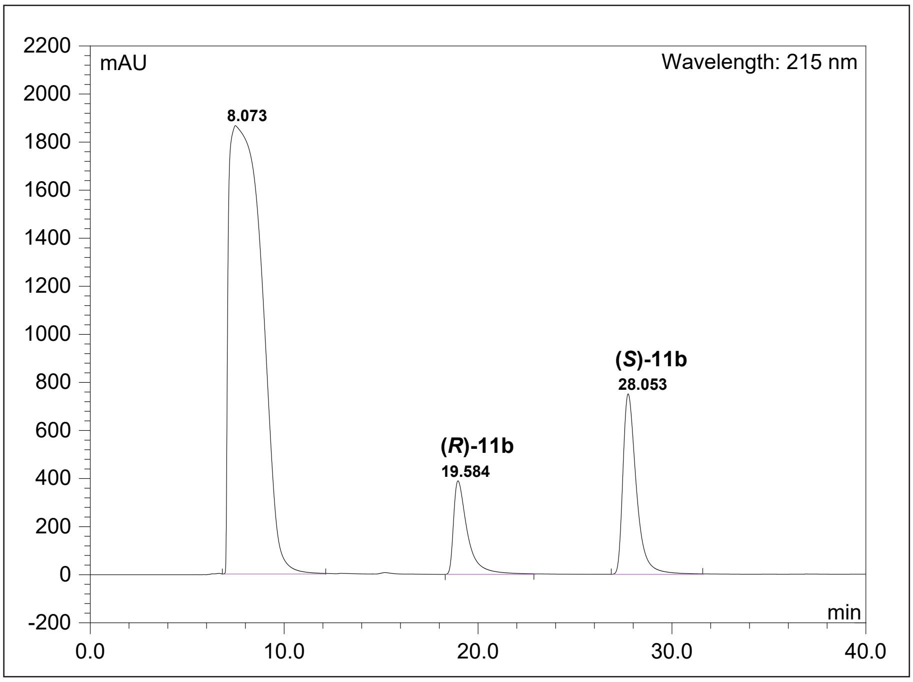

Figure S30. HPLC analysis for determination of enantiomeric excess of 11b: column OB-H; flow rate: 0.8 ml/min; Mobile phase hexane/isopropanol (***v***/***v***) = 98:2; (*R*)-11b = 19.6 min; (*S*)-11b =28.1 min.

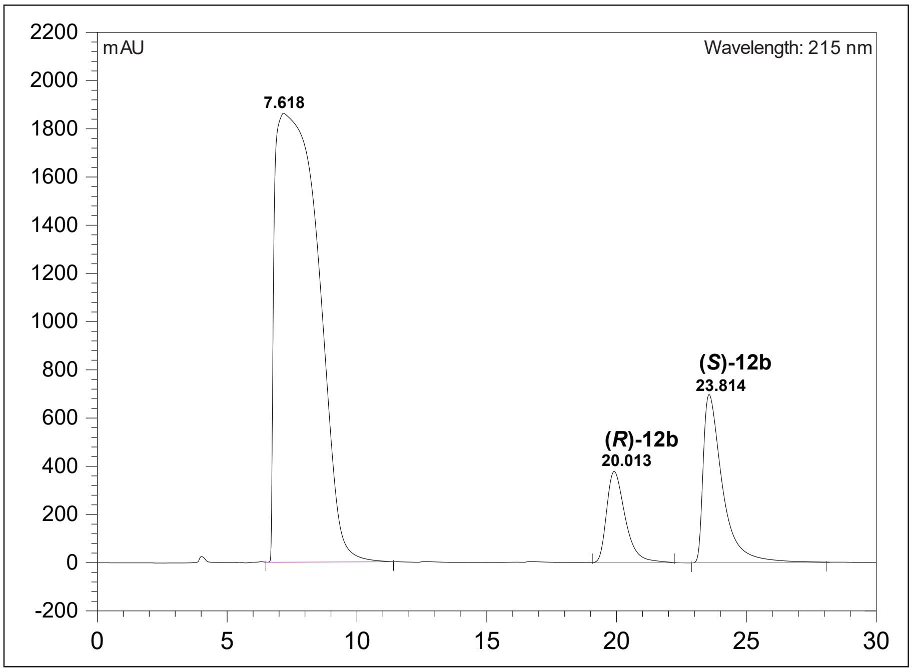

Figure S31. HPLC analysis for determination of enantiomeric excess of 12b: column OB-H; flow rate: 0.8 ml/min; Mobile phase hexane/isopropanol (***v***/***v***) = 98:2; (*R*)-12b = 20.0 min; (*S*)-12b = 23.8 min.

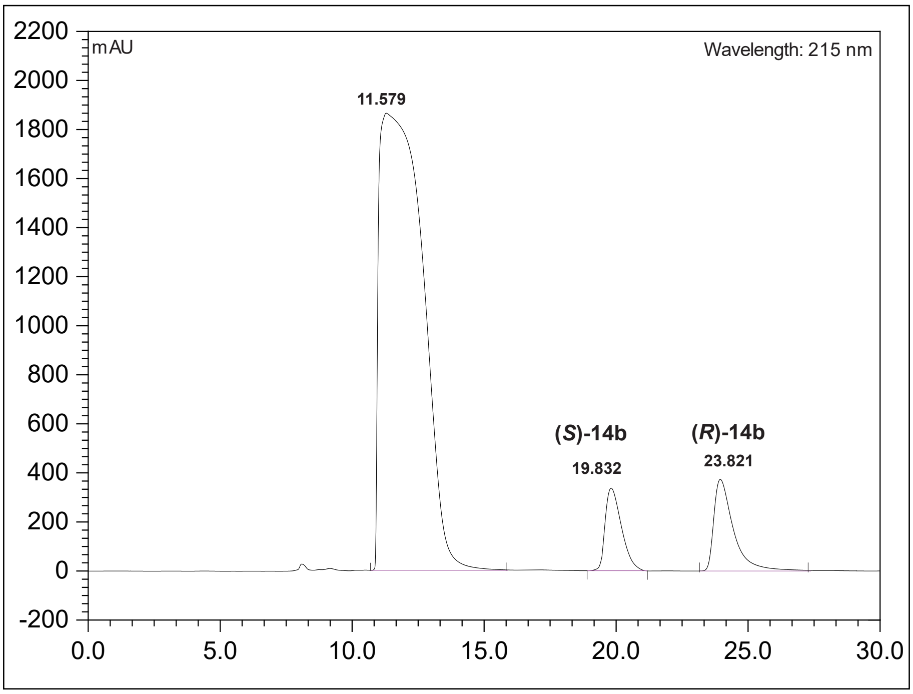

Figure S32. HPLC analysis for determination of enantiomeric excess of 14b: column OB-H; flow rate: 0.4 ml/min; Mobile phase hexane/isopropanol (***v***/***v***) = 9:1; (*R*)-12b = 23.9 min; (*S*)-12b = 19.8 min.

Table S1. Secondary structure contents (%) of WT SCRII, SCRII-mtf and crosslinked SCRII.[a]

Enzymes	α-helix	β-strand	β-turn	random coil
WT SCRII	31.0	20.4	20.6	28.0
SCRII-mtf	31.2	20.2	20.7	27.9
Crosslinked SCRII	31.8	20.5	19.9	27.8

[a] The contents were determined by the deconvolution of circular dichroism (CD) data using K2d method available in the Dichroweb server.

Table S2 Michaelis-Menten and Lineweaver-Burk plots of WT SCRII, SCRII-mtf and crosslinked SCRII towards **1a-14a.**

S	WT SCRII	SCRII-mtf	Crosslinked SCRII

1a

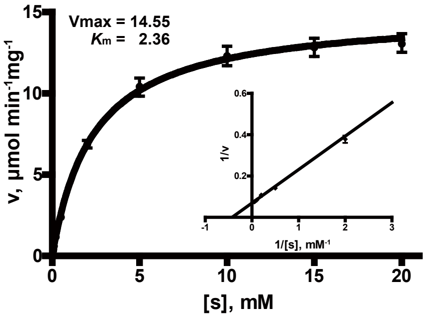

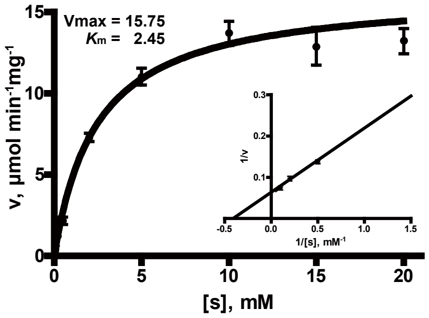

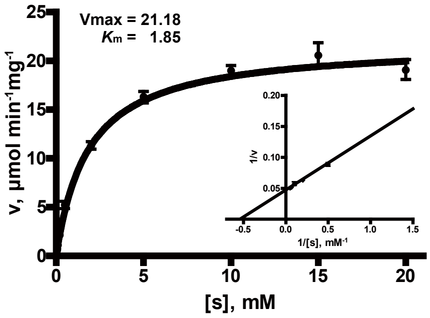

2a

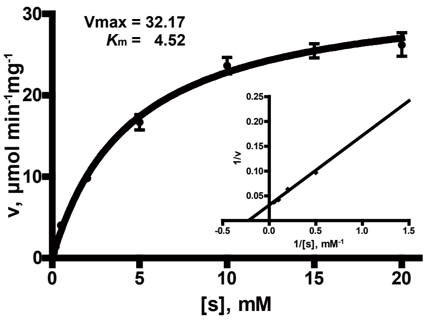

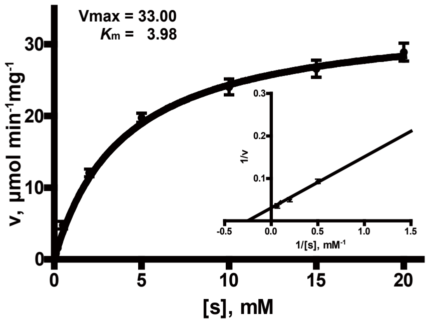

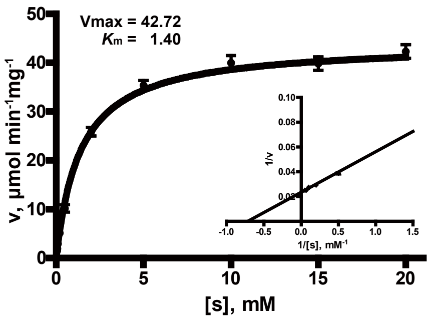

3a

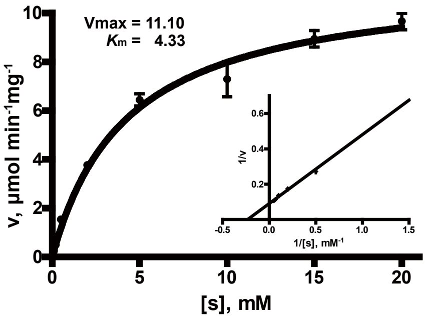

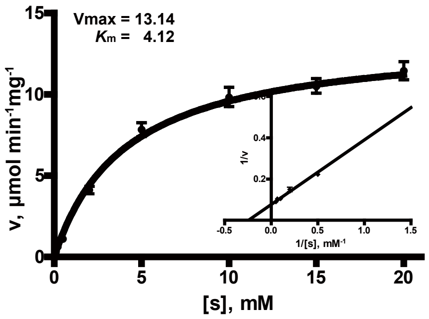

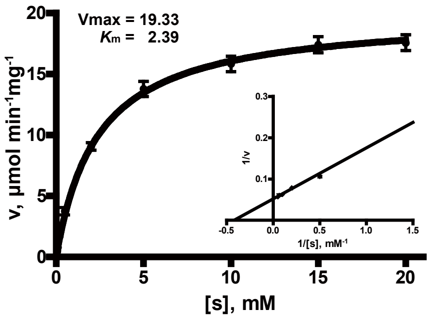

4a

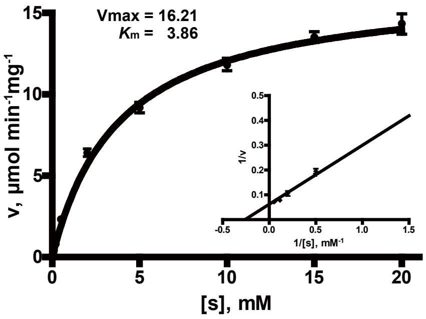

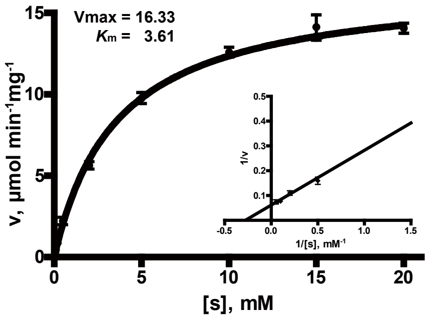

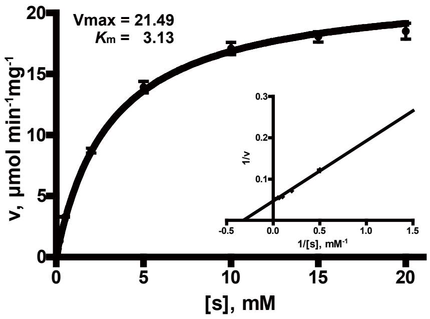

5a

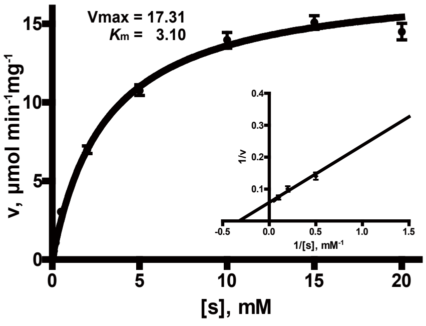

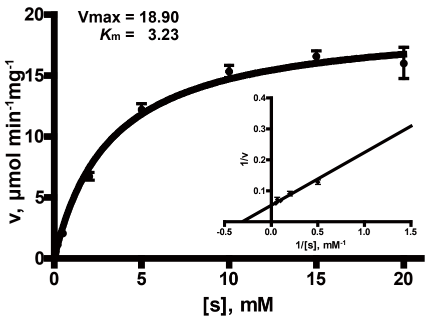

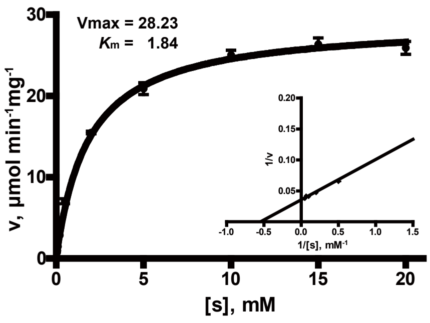

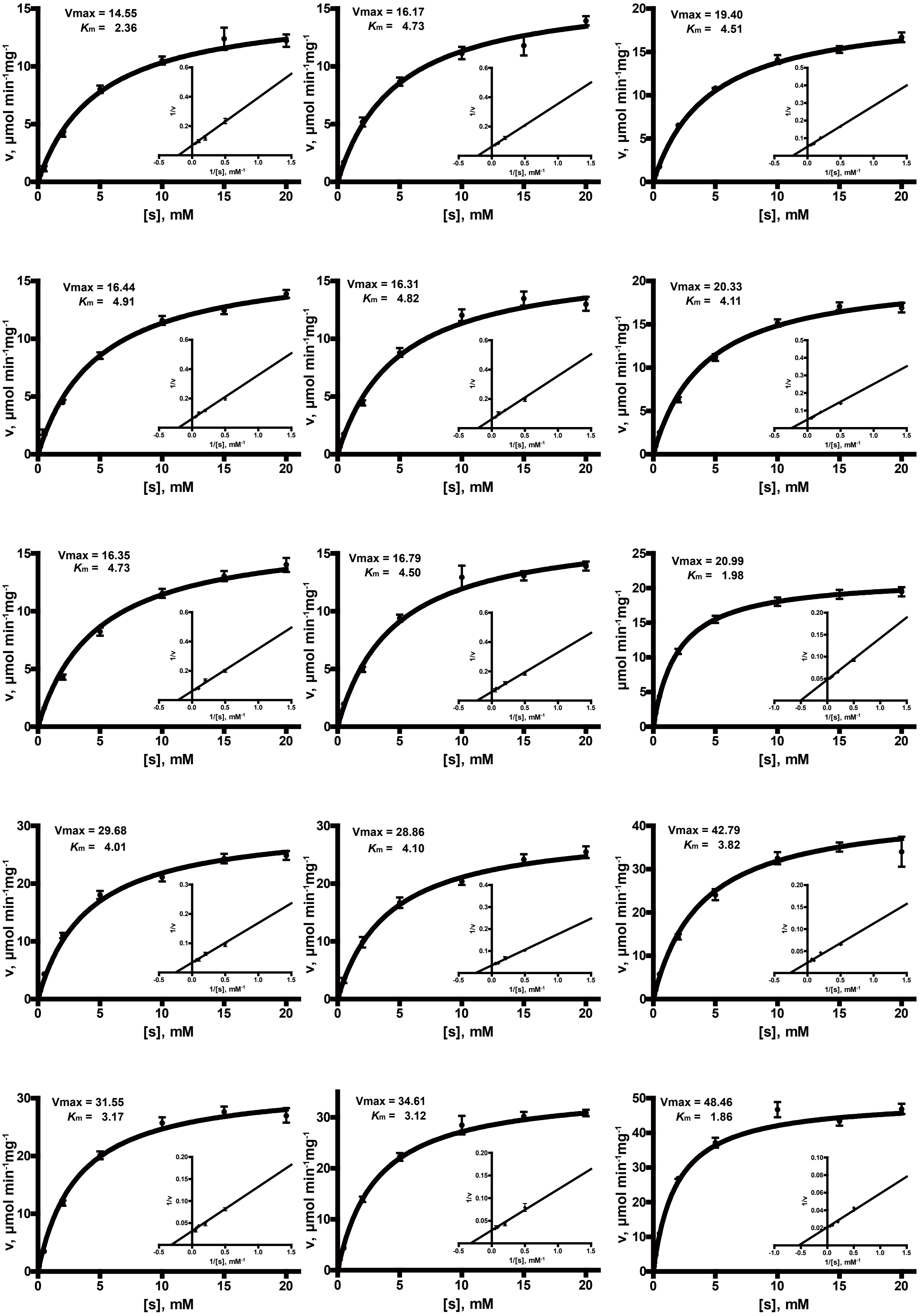

7a

8a

9a

10a

11a

12a

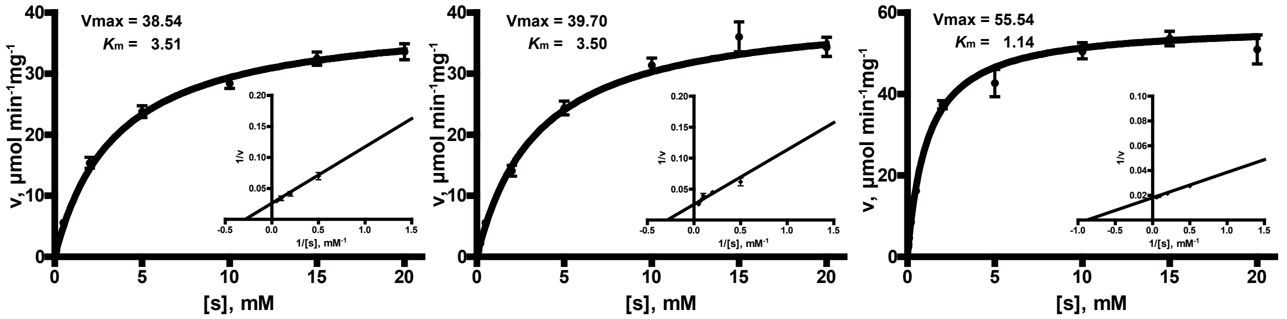

Vmax = 38.54
Km = 3.51
Vmax = 39.70
Km = 3.50
Vmax = 55.54
Km = 1.14
v, μmol min-1mg-1
[s], mM
1/v
1/[s], mM-1

14a

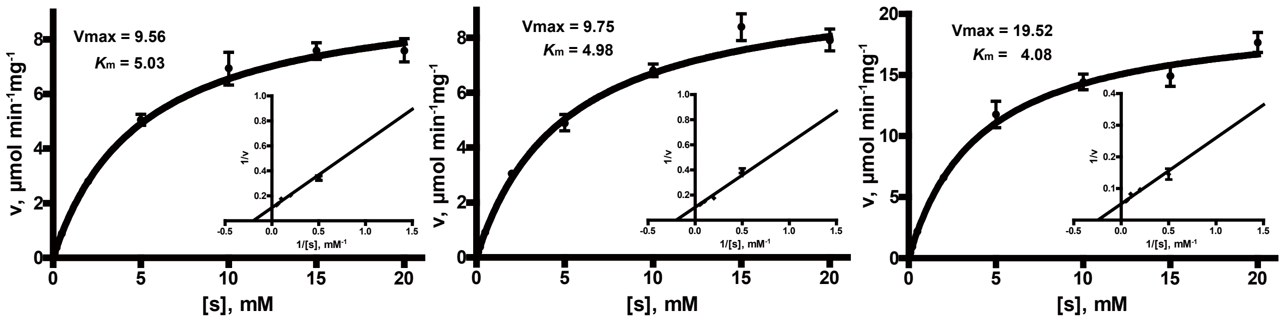

Vmax = 9.56
Km = 5.03
Vmax = 9.75
Km = 4.98
Vmax = 19.52
Km = 4.08
v, μmol min-1mg-1
[s], mM
1/v
1/[s], mM-1

Table S3. Primers in this work and PCR thermal cycle detail.[a]

Primers	Sequence (5'→3')	Thermal cycle detail
adhr-mtf_F	CCCATATG**GGAGGCGGA**ACTGATCGTTTAAAAG	98 °C for 30 s; 98 °C for 10 s, 50 °C for 15 s, 72 °C for 40 s for 30 cycles; 72 °C for 10 min.
adhr-mtf_R	CCCTCGAG**GCCGCCGGTTTCCGGAAGGCTGCCACCGCCACC**TTGAGCAGTGTATCCACC	
*c*1-mtf_F	CGGGATCCATG**GGAGGCGGA**TCACTTGCTGGAAAAG	98 °C for 30 s; 98 °C for 10 s, 55 °C for 15 s, 72 °C for 50 s for 30 cycles; 72 °C for 10 min.
*c*1-mtf_R	CGGAATTC**GCCGCCGGTTTCCGGAAGGCTGCCACCGCCACC**ATCATACTTGGAATACTCTTTCG	
*c*2-mtf_F	CGGGATCCATG**GGAGGCGGA**ACTCAAAGTAACTTAC	98 °C for 30 s; 98 °C for 10 s, 45 °C for 15 s, 72 °C for 50 s for 30 cycles; 72 °C for 10 min.
*c*2-mtf_R	CCCTCGAG**GCCGCCGGTTTCCGGAAGGCTGCCACCGCCACC**CAAATCTTTAAATTGCTC	
*cr*2-mtf_F	CGGGATCCATG**GGAGGCGGA**ACATTTACAGTGGTG	98 °C for 30 s; 98 °C for 10 s, 50 °C for 15 s, 72 °C for 55 s for 30 cycles; 72 °C for 10 min.
*cr*2-mtf_R	CGGAATTC**GCCGCCGGTTTCCGGAAGGCTGCCACCGCCACC**CCCACGGTACGC	
*cr*4-mtf_F	CGGGATCCATG**GGAGGCGGA**ACGTTTCAGCATTTTTTAAG	98 °C for 30 s; 98 °C for 10 s, 55 °C for 15 s, 72 °C for 45 s for 30 cycles; 72 °C for 10 min.
*cr*4-mtf_R	CCCTCGAG**GCCGCCGGTTTCCGGAAGGCTGCCACCGCCACC**AACGCAAGTGTACCCACC	
*s*1-mtf_F	CGGGATCCATG**GGAGGCGGA**GCTAAGAACTTCTCC	98 °C for 30 s; 98 °C for 10 s, 50 °C for 15 s, 72 °C for 45 s for 30 cycles; 72 °C for 10 min.
*s*1-mtf_R	CGGAATTC**GCCGCCGGTTTCCGGAAGGCTGCCACCGCCACC**GGGAAGCGTGTAGC	
scr1-mtf_F	CGGGATCCATG**GGAGGCGGA**AGTAAAGACGAAACAATTTC	98 °C for 30 s; 98 °C for 10 s, 48 °C for 15 s, 72 °C for 60 s for 30 cycles; 72 °C for 10 min.
scr1-mtf_R	CCCTCGAG**GCCGCCGGTTTCCGGAAGGCTGCCACCGCCACC**TGGGACAGTATAACCAC	
*scr*II-mtf_F	CCCATG**GGC**GAAATCGAATC	98 °C for 30 s; 98 °C for 10 s, 55 °C for 15 s, 72 °C for 45 s for 30 cycles; 72 °C for 10 min.
*scr*II-mtf_R	CCCTCGAG**GCCGCCGGTTTCCGGAAGGCTGCCACCGCCACC**TGGACAAGTGTAACCACCATC	
*scr*3-mtf_F	CCCATATG**GGAGGCGGA**GGCGAAATCGAATC	98 °C for 30 s; 98 °C for 10 s, 52 °C for 15 s, 72 °C for 45 s for 30 cycles; 72 °C for 10 min.
*scr*3-mtf_R	CCCTCGAG**GCCGCCGGTTTCCGGAAGGCTGCCACCGCCACC**TGGACAGGTGAATCCAC	
*srt*A_F	CGCCATATGCAAGCTAAACCTC	98 °C for 30 s; 98 °C for 10 s, 50 °C for 15 s, 72 °C for 25 s for 30 cycles; 72 °C for 10 min.
*srt*A_R	CCGCTCGAGTTTGACTTCTGTAGCTAC	

[a] The N-terminal $(Gly)_n$ and C-terminal GGGGSLPETGG motif are corresponding to bold DNA sequences.
